# Advances of Self-Healing Polymers Incorporated in Perovskite Solar Cells for High Durability

**DOI:** 10.1007/s40820-026-02087-x

**Published:** 2026-03-04

**Authors:** Jialiang Li, Mengqi Geng, Le Jiang, Tingting Xu

**Affiliations:** 1 School of Chemistry and Chemical Engineering, Northwestern Polytechnical University, Xi’an, 710129 Shaanxi People’s Republic of China; 2 Shenzhen Research Institute of Northwestern Polytechnical University, Shenzhen, 518057 Guangdong People’s Republic of China

**Keywords:** Perovskite solar cells, Self-healing polymers, Multiple dynamic bonds, Self-healing mechanism, Chemical or physical damage

## Abstract

Perovskite solar cells (PSCs) have achieved remarkable power conversion efficiencies (PCE) exceeding 27%, while their operational instability under environmental stress (e.g., moisture, heat, mechanical bending) remains a critical barrier to commercialization. Self-healing polymers (SHPs) with dynamic covalent bonds or non-covalent bonds have emerged as an innovative solution to enhance the durability of PSCs through autonomous damage healing. Although SHPs have been proved to be quite promising for enhance the reliability of PSCs, there is still lacking systematic molecular design strategies tailored for practical cooperation SHPs with versatile types of PSCs. Herein, this review systematically organizes the recent research progress of self-healing PSCs from the perspective of application-oriented design principles. The self-healing mechanisms of PSCs using SHPs under chemical and mechanical damage modes are first comprehensively explored, and a multi-dimensional self-healing evaluation system is proposed. Subsequently, the distinct effects of SHPs as additives, interfacial modifiers, and encapsulation materials in PSCs are summarized. More importantly, the incorporation methods of SHPs in PSCs and the structural characteristics of representative SHPs are systematically analyzed, with application-specific design principles for optimized performance proposed. Finally, the challenges and opportunities in the optimization of self-healing material properties, in situ characterization techniques, and scalable fabrication are outlined. This work aims to facilitate the transition of SHP-based self-healing PSCs from laboratory research to real-world applications, providing a roadmap for future developments in this emerging field.

## Highlights

- Examining recovery mechanisms through self-healing polymer–perovskite interactions under chemical/mechanical stress.
- Proposing a multi-scale protocol combining electrical, morphological, and chemical metrics to quantitatively assess healing efficacy.
- Establishing a systematic design strategy based on healing stimuli and device integration scenarios, linking molecular features to functional outcomes.

## Graphical abstract

**Figure:**
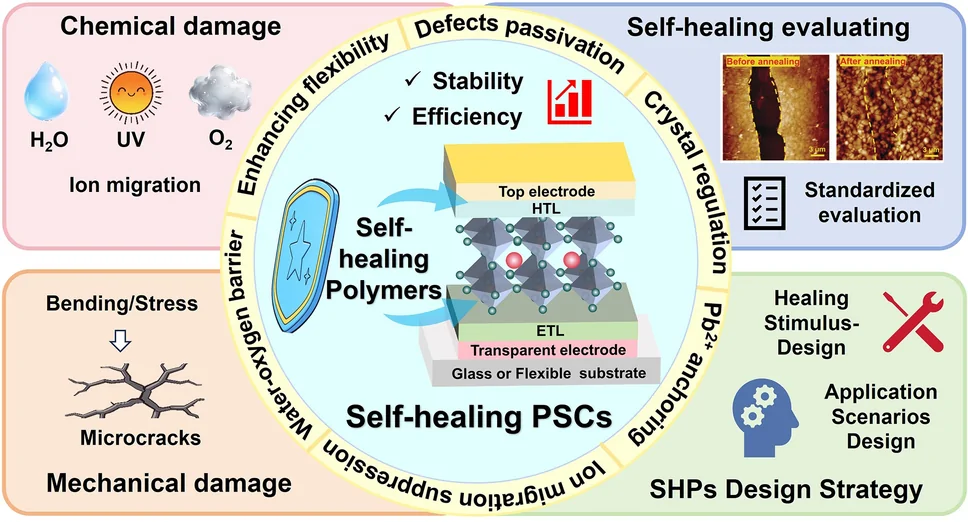

## Introduction

With the advancement of renewable energy and electronic technology, many electronic devices have gradually become an integral part of daily life due to their portability and multifunctionality [1]. Energy sources as the core component in various electronic devices make them functional properly [2]. Perovskite solar cells (PSCs), characterized by their high-power conversion efficiency (PCE), lightweight, and flexible properties, demonstrate significant potential in wearable electronic applications [3]. Since the first PSC reported in 2009, over a decade of intensive research has enabled certified PCEs to surge from 3.8% to the current 27.3% [4, 5], achieving comparable PCE performance to the crystalline silicon solar cells developed over 70 years. To date, researchers have dedicated to exploring more rational structural designs to approach the Shockley–Queisser (SQ) theoretical efficiency limit (33.7%) [6]. Enhancing stability and operational lifespan of PSCs to accelerate commercialization has become more critically important than competing with the rising race of PCE [7]. In practical applications, perovskite materials, due to their ionic crystal characteristics and soft lattice nature [8], are prone to ion migration [9], phase segregation [10], microcrack formation [11], and interface degradation under light, humidity, thermal stress, mechanical strain, or electric fields [12]. These dynamic degradations accumulate over time, exacerbating non-radiative recombination, impeding carrier transport, and triggering stability collapse [13]. These unstable factors under various conditions have hindered further commercialization of PSC technology. Particularly, once damage occurs in PSCs under extreme environmental conditions, irreversible performance degradation ensues.

To address these challenges, extensive research have employed polymeric strategies to enhance the performance and stability of PSCs [14]. It has been proved that traditional polymers such as poly(methyl methacrylate) (PMMA) [15], polydimethylsiloxane (PDMS) [16], polyisobutylene (PIB) [17], and epoxy resin [18] have shown significant advantages in PSCs. However, their static chemical structure is still difficult to cope with the inevitable mechanical stress and chemical erosion of devices in long-term operation [19, 20]. Thus, the above challenge has led to innovative applications of self-healing polymers (SHPs). SHPs with the structure advantages of dynamic bonds can get repaired under mechanical force [21]. SHPs hold promise for mitigating performance losses or even restoring original functionality in PSCs subjected to mechanical damage, thereby significantly enhancing device stability, operational lifespan, and reducing environmental pollution. Studies reveal that the tunable chemical structures of these SHPs enable multifunctional roles through rational molecular design [22]. Since 2016, a series of SHPs such as self-healing polyurethane (PU), and poly(lipoic acid) have been attempted for enhancing performance and stability of PSCs. Beyond autonomous repair, they enhanced perovskite film flexibility, regulated crystallization processes, blocked moisture/oxygen ingress, passivated defects, suppressed ion migration, and sequester lead ions, etc. [23, 24]. These synergistic effects of SHPs not only improve PSC performance, but also significantly boost device stability, offering a valuable solution for advancing perovskite photovoltaic technology [25, 26].

**Fig. 1:**
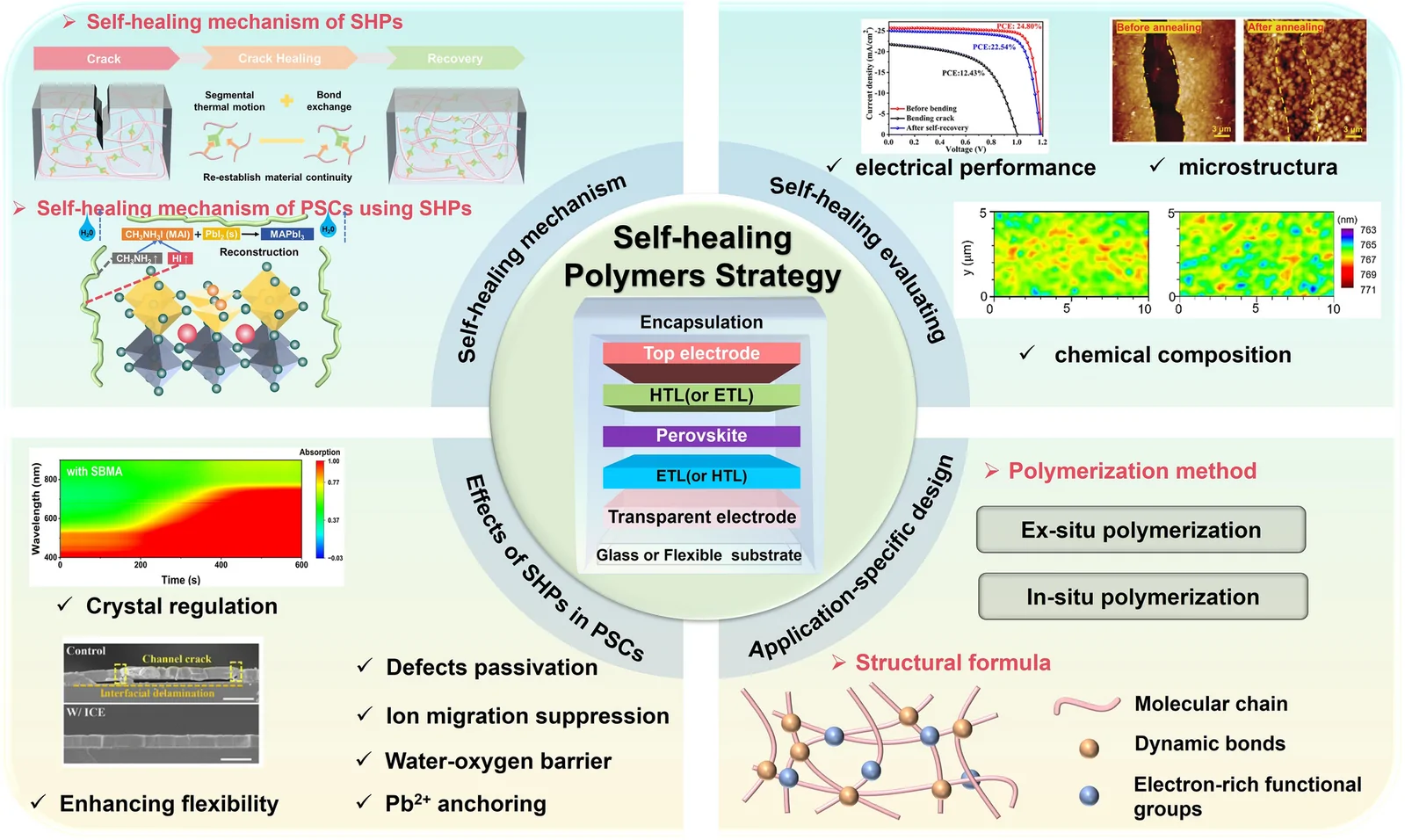
Schematic illustration of SHPs strategy in PSCs summarized in this work

While several reviews have highlighted advances in the implementation of functional polymers in PSCs, existing summaries have predominantly focused on the self-healing mechanisms of the polymers themselves, paying less attention to how SHPs interact with perovskite materials to achieve recovery. This review offers an innovative perspective by re-examining the healing mechanisms through the interactions between SHPs and perovskites. Furthermore, it suggests a novel assessment for self-healing efficacy and summarizes rational design principles for SHPs. As shown in Fig. 1, a schematic illustration of the self-healing polymer strategy for PSCs is demonstrated in this review. In order to advance mild self-healing strategies in PSCs, the repair mechanisms of SHPs and the application of multiple dynamic bonds are introduced. The self-healing mechanisms of PSCs under chemical and mechanical damage modes are systematically reviewed. Multi-scale evaluation methods for self-healing performance are proposed, including macroscopic device performance testing, microscopic structure, and morphology analysis, as well as chemical composition and structural characterization. Depending on the functionality of the SHPs in PSC, SHPs applied for additive engineering, interface engineering, and device encapsulation are highlighted. Besides, SHPs for real application particularly the ex-synthesized and in situ synthesized SHPs with different configurations of PSCs are discussed. Several typical SHPs such as step-growth polymers, ring-opening polymers, addition polymers, and copolymers are summarized and emphasized on their structure–property relationships tailored for photovoltaic durability. Moreover, application-specific design principles for optimizing performance are proposed. Finally, this review systematically addresses the key challenges and future directions, including elucidating fundamental healing mechanisms and establishing long-term reliability protocols, designing polymers that balance self-healing, passivation, and conductivity, enabling scalable fabrication and roll-to-roll compatibility, and pioneering data-driven development through machine learning, thereby bridging the critical gap between laboratory innovation and industrial application.

## Self-Healing Mechanism and Performance Evaluation of PSCs

PSCs based on SHPs possess inherent self-healing capabilities, which largely originate from the intrinsic self-healing properties of polymers. Thus, this section will firstly provide an overview of the self-healing mechanisms of polymers and then discuss the interactions of SHPs with perovskite materials. The incorporation of these two materials demonstrates much enhanced response under chemical or physical damage. Based on these two damage origins, how their interactions with perovskites enable repair responses to two primary damage mechanisms is specifically examined: mechanical fractures and chemical degradation. Additionally, the experimental approaches to evaluate self-healing performance and current evaluation methodologies for assessing self-healing performance are discussed in this field.

### Self-Healing Mechanism of SHPs Materials

**Fig. 2:**
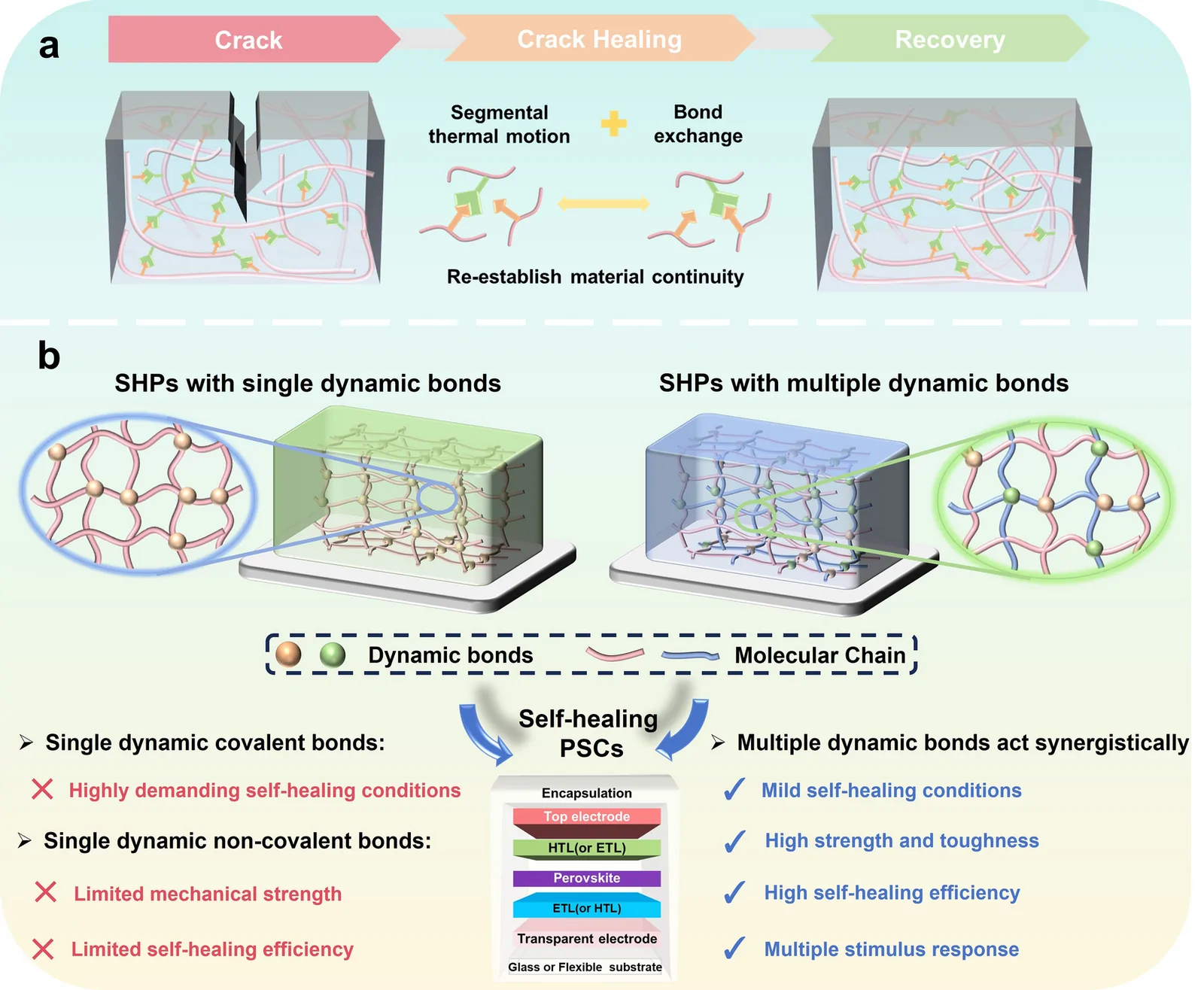
**a** Schematic diagram of self-healing process; **b** Schematic diagram of multiple dynamic bonds of SHPSs

The self-healing mechanism of polymers fundamentally arises from the synergistic interplay between the thermal motion of molecular segments and the reversible cleavage/reformation of dynamic bonds [27]. As illustrated in Fig. 2a, upon crack initiation, dynamic bonds at the fracture interface undergo cleavage. When the temperature exceeds the glass transition temperature (*T*_g_), molecular segments become activated and gain mobility through conformational changes—primarily involving rotation around C–C single bonds—and chain slippage. This enhanced mobility enables polymer chains to diffuse toward the damaged region (e.g., a crack) [28]. The diffusion process is governed by Fick’s laws [29]. Driven by a concentration gradient, chains from opposing crack surfaces migrate into the fracture zone, where interdiffusion leads to entanglement and reconstruction of a physically cross-linked network that bridges the gap [30]. The self-healing dynamics are governed by the timescales associated with bond rearrangement and chain relaxation. The characteristic time for bond reformation, *τ*_bond_, can be described by an Arrhenius-type relationship [31]:

1 $$\tau_{{{\mathrm{bond}}}} = \tau_{0} \times \exp \left( {\frac{{E_{a} }}{{{\mathrm{RT}}}}} \right)$$

where *E*_a_ is the activation energy for bond recombination, *R* is the universal gas constant, *T* is the absolute temperature, and τ₀ is the pre-exponential factor [31]. At temperatures above *T*_*g*_, the increased thermal energy not only facilitates chain mobility, but also reduces τ_bond_, thus accelerating the healing kinetics. Concurrently, the thermal motion of molecular segments brings the crack interfaces into close proximity, enabling dynamic bonds to reform once the activation energy barrier is surmounted under thermal or other external stimuli [32].

These bond exchange reactions regenerate cross-linking sites. The reorganized dynamic network fills the crack void, while the underlying covalent network provides structural confinement to prevent macroscopic collapse [33, 34]. Throughout this process, ongoing molecular thermal motion and conformational relaxation of chains release internal stresses, ultimately restoring mechanical equilibrium in the healed material. In the overall self-healing dynamics, reversible bonding constitutes the chemical foundation of repair, which fundamentally involves the reversible breaking and reformation of bonds. Based on bonding characteristics, these can be classified into two categories: dynamic covalent bonds (chemical bonds) and dynamic non-covalent bonds (physical bonds) [35, 36].

Dynamic covalent bonds arise from the sharing of electron pairs between atoms with moderate bond energy, generally between those of traditional covalent bonds (e.g., C–C bonds, ~ 350 kJ mol^−1^) and non-covalent bonds. A key feature is the tunability of bond energy and the reversible design of reaction pathways, enabling selective bond cleavage and reformation under specific stimuli [37–39]. This mechanism typically imparts higher mechanical strength to materials, though the kinetics are often relatively sluggish and requires energy input [40, 41]. For instance, the repair process based on the Diels–Alder (DA) reaction depends on heating–cooling cycles, generally requiring elevation to high temperatures (e.g., 90–130 °C) and maintenance for a certain duration to achieve sufficient bond exchange and network reorganization [42]. Disulfide bond exchange usually occurs at moderate temperatures (~ 60 °C) [43]. Owing to their lower bond energy, diselenide bonds exhibit faster exchange kinetics and can even be triggered by visible light at room temperature, enabling more rapid repair [44]. The reversibility of dynamic imine bonds and boronic ester bonds is strongly influenced by pH and moisture. For example, imine bonds (C=N) are prone to hydrolytic cleavage under acidic conditions but can reform in neutral or alkaline environments [45].

Dynamic non-covalent bonds are reversible bonding networks formed through interactions such as hydrogen bonding, metal–ligand coordination, or *π*–*π* stacking. These bonds exhibit lower bond strength (typically 5–50 kJ mol^−1^), facilitating rapid dissociation and reconfiguration under mild conditions [46, 47]. They enable multiple cycles of autonomous self-healing on timescales ranging from seconds to minutes. For instance, the association and dissociation of hydrogen bonds represent a rapid dynamic equilibrium process, allowing instantaneous rearrangement and fast self-repair at room temperature [48]. In addition, self-healing kinetics based on metal–ligand coordination, host–guest interactions, and ionic interactions can also proceed spontaneously at ambient temperature, generally without requiring external stimuli [49, 50]. This property enables room-temperature self-healing and enhances the versatility of polymers in applications such as PSCs.

Beyond single dynamic bond systems in SHPs for PSCs, multiple dynamic bonds have gained prominence due to the limitations of single-bond systems. As depicted in Fig. 2b, for instance, dynamic covalent bonds such as disulfide linkages often require prolonged exposure to harsh conditions (e.g., elevated temperatures or UV irradiation) to initiate self-healing [51]. Meanwhile, systems based solely on dynamic non-covalent bonds such as hydrogen bonds often suffer from inadequate mechanical robustness and suboptimal healing efficiency [52]. These limitations have prompted a shift toward multi-dynamic bond systems that operate synergistically, particularly under mild, market-adaptable conditions.

The synergistic effect of multiple dynamic bonds involves the integration of two or more types of reversible bonds (e.g., dynamic covalent and non-covalent bonds) within a single material [53]. By leveraging their complementary bond strengths, stimulus responsiveness, and reorganization kinetics, this strategy enhances key material properties, including self-healing efficiency, mechanical adaptability, and environmental stability [54]. Such multi-dynamic bonding systems overcome the functional constraints of single-bond systems. The strategic combination of bond types—such as hydrogen bonds, metal–ligand coordination, and dynamic covalent bonds—enables hierarchical control over material performance: (1) mechanical integrity via covalent reinforcement, (2) tunable self-healing kinetics through reversible non-covalent bonds, (3) solution processability afforded by bond lability, and (4) tailored environmental responsiveness through selective bond activation.

### Self-Healing Mechanism of PSCs Using SHPs

The integration of SHPs with perovskite devices represents an innovative strategy for enhancing performance and addressing stability challenges in PSCs. This approach relies on the intrinsic capability of the material to autonomously recover its structure and function following damage. This section elucidates elucidating the specific molecular mechanisms by which SHPs, when incorporated into PSCs, trigger and enable self-healing in response to both chemical and mechanical damage modes. Understanding these underlying mechanisms is critical for the rational design of SHPs and the optimization of high-efficiency self-healing PSCs.

#### Self-Healing Mechanism Under Chemical Damage Mode

Chemical degradation of perovskite materials, particularly halide perovskites such as MAPbI_3_ and FAPbI_3_, represents a core challenge to the stability of PSCs. Based on degradation triggers, chemical degradation primarily encompasses two categories: (1) chemical decomposition of the perovskite material induced by environmental factors (moisture, oxygen, heat, and ultraviolet light) and (2) defects arising from ion migration within the perovskite lattice. Specific manifestations include the generation of the yellow PbI_2_ phase, volatilization/decomposition of organic cations (e.g., MA^+^, FA^+^), loss of halide ions (I^−^), and the formation of detrimental compounds at interfaces (e.g., metallic Pb^0^) [55–57].

**Fig. 3:**
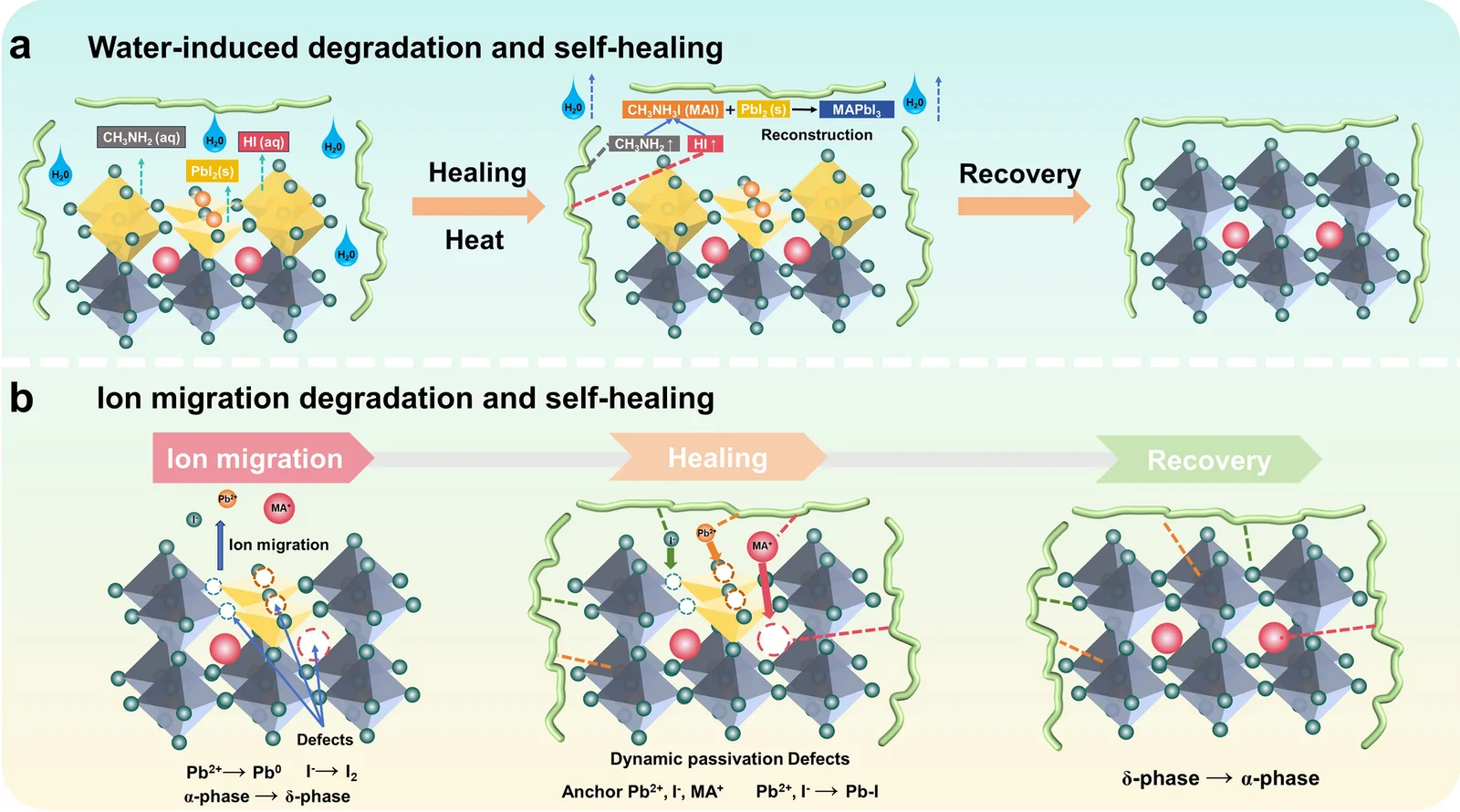
**a** Schematic diagram of water-induced degradation and self-healing; **b** Schematic diagram of ion migration degradation and self-healing

The introduction of SHPs has been demonstrated to effectively repair such damage. The essence of this repair lies in the specific chemical interactions and dynamic characteristics between functional groups within the SHPs and the perovskite material. Taking water-induced MAPbI_3_ degradation as an example (Fig. 3a), the decomposition produces volatile CH_3_NH_2_ and HI, while methylammonium iodide (MAI) can only reform if HI is captured [58]. Functional groups (e.g., carbonyl, amino) in the polymer preferentially coordinate with free Pb^2+^ to form stable complexes, inhibiting PbI_2_ aggregation [59–61]. Oxygen/nitrogen-containing groups also adsorb CH_3_NH_2_ and HI via hydrogen bonding or electrostatic interactions, confining the ions within the polymer network. Under self-healing conditions (e.g., heating), the dynamic non-covalent bonds allow the confined ions to rearrange [62]. The rearranged ions, along with newly formed MAI, diffuse within the flexible polymer network and recombine to form MAPbI_3_ nuclei. Functional groups in the self-healing polymer can act as templates, guiding the regrowth of MAPbI_3_ along specific crystal planes, thereby restoring the optoelectronic properties of the PSCs [63]. Research has confirmed that the mechanism of water-induced perovskite chemical degradation repaired by SHPs is also applicable to other systems, such as those based on formamidinium (FA). Bis-MPA-NHBoc dendrimer (NHD) as an additive during perovskite film formation successfully enables the reversible capture and release of volatile components (FA) [64]. This mechanism facilitates the restoration of 90% of the initial PCE after 10 cycles of alternating exposure to high-humidity (60 ± 1 °C, 85 ± 5% RH) and dry (25 ± 1 °C, < 20% RH) conditions.

To date, few studies have specifically investigated the self-healing mechanisms following chemical degradation of perovskites induced solely by oxygen, heat, or ultraviolet (UV) light. This represents a significant area warranting future in-depth research. Furthermore, the incorporation of SHPs inherently provides an effective barrier against moisture and oxygen ingress. In majority of studies, where SHPs restore the performance decay and device efficiency of PSCs, the improvement primarily stems from the mitigation of internal factors—namely ion migration and defect formation—occurring during device operation. Ion migration—triggered by electric fields, light, or heat—drives species such as Pb^2^^+^, MA^+^, and I⁻ from their lattice sites to interfaces. At the anode or hole transport layer (HTL), migrating I⁻ can be oxidized to I_2_, disrupting the perovskite structure and creating halide vacancies. Concurrently, at the cathode or electron transport layer (ETL), these vacancies facilitate the reduction of Pb^2+^ to metallic Pb^0^ [65–67]. This cascade induces defect aggregation, lattice distortion, microcracking, or phase separation, including the transition from the photoactive α-phase to the non-photoactive *δ*-phase in FAPbI_3_ [68]. As shown in Fig. 3b, SHPs incorporating Lewis base groups (e.g., carbonyl, amino, phosphine oxide) or anionic groups (e.g., sulfonate, carboxylate) can mitigate this degradation by capturing migrating I⁻ ions through specific interactions [69]. This effectively immobilizes ions directly involved in decomposition reactions, thereby interrupting the reaction chain. Upon activation of the self-healing mechanism, enhanced polymer chain mobility facilitates the guided diffusion of neighboring I⁻ ions to fill vacancies, restoring Pb-I bonds. For chemical damage originating from defects, a similar principle applies: Driven by segmental motion, the self-healing polymer migrates to newly exposed defect sites (e.g., Pb^2+^ vacancies, I⁻ vacancies), enabling continuous dynamic passivation of nascent defects and reducing non-radiative recombination.

For PSCs undergoing chemical degradation modes, SHPs function primarily through an anchoring effect, mediated by interactions between their functional groups and the perovskite material. The dynamic bonding inherent to these polymers, coupled with molecular thermal motion, facilitates persistent dynamic interactions with the perovskite lattice. This synergistic mechanism underpins the self-repair capability of PSCs.

#### Self-Healing Mechanism Under Mechanical Damage Mode

The mechanical degradation mechanism of flexible perovskite solar cells (F-PSCs) following repeated bending stems from the brittle fracture of the perovskite layer and interfacial delamination caused by stress mismatch between layers [70, 71]. The perovskite absorber layer, intrinsically brittle, exhibits high hardness but low fracture toughness and weak tensile strength, rendering it susceptible to crack formation under tensile stress. Stress concentration occurs at grain boundaries, defects within the perovskite, and layer interfaces, initiating microcracks [72, 73]. Under cyclic bending loads (fatigue), these microcracks propagate and coalesce, ultimately forming macroscopic through-thickness cracks and interfacial delamination. Microcracks nucleating and propagating within polycrystalline perovskite films not only directly sever carrier transport pathways and increase series resistance, but also significantly promote the proliferation of halide vacancy defects, thereby intensifying non-radiative recombination [74]. Concurrently, cracks extending to functional layer interfaces can induce delamination, leading to interfacial contact failure, reduced carrier extraction efficiency, and the creation of channels for moisture and oxygen infiltration, which collectively accelerate the chemical degradation of the material [75]. Such structural damage and electrical performance degradation are coupled together, ultimately manifesting as a sharp decline in fill factor and short-circuit current density [76].

**Fig. 4:**
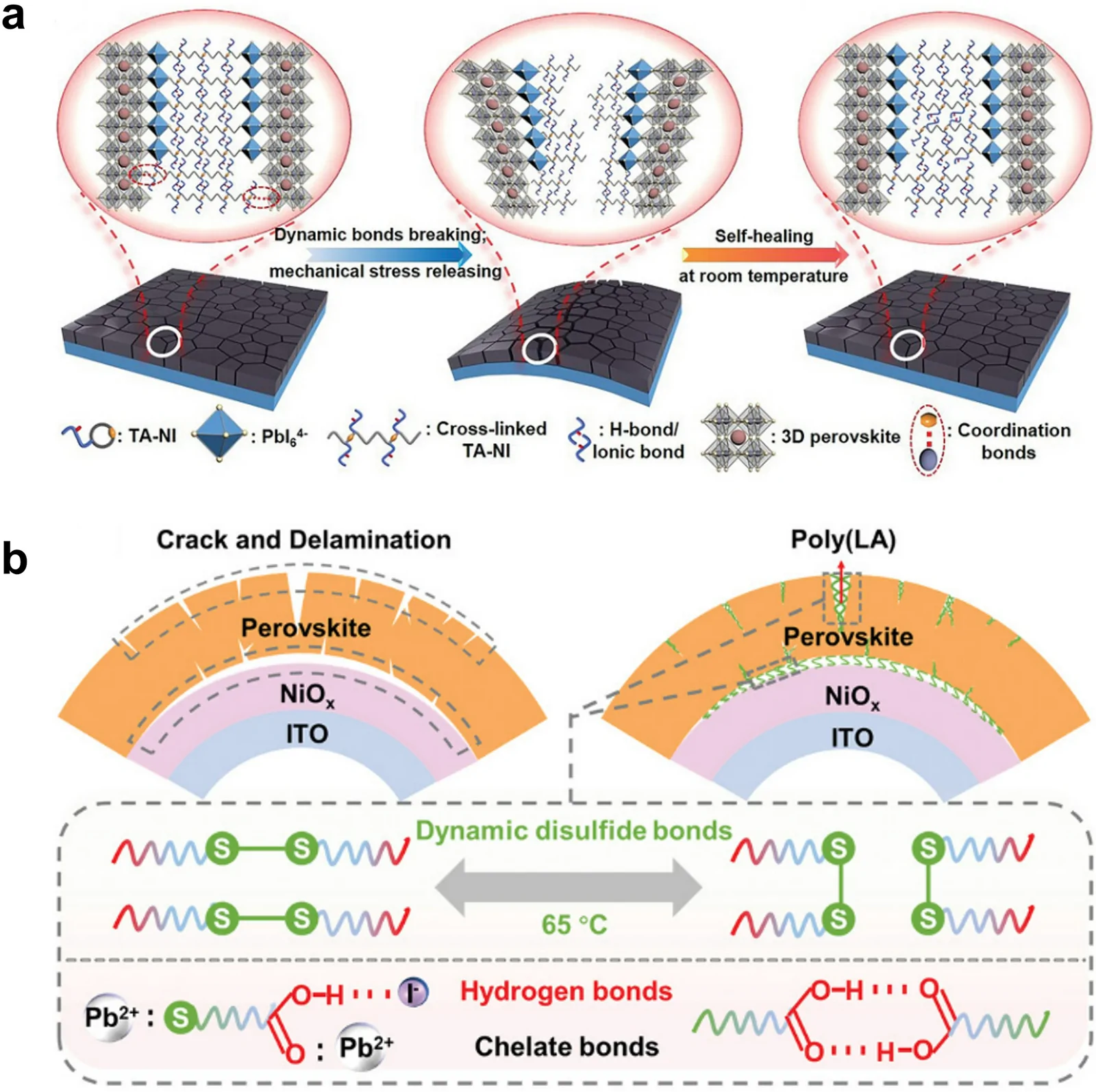
**a** Schematic diagram of self-healing process of TA-NI [77] Copyright 2023, Wiley–VCH; **b** Schematic diagram of the cracks and delaminations caused by mechanical bending, as well as the self-healing effect of the optimized device assisted by dynamic cross-linked poly(LA) [78] Copyright 2024, Wiley–VCH

The application of SHPs as interfacial engineering layers or additives within F-PSCs enables the self-repair of optoelectronic properties after mechanical failure induced by repeated bending. The underlying mechanism involves: Upon microcrack formation in the perovskite layer, the SHP, activated by an external stimulus (e.g., heat, light), undergoes localized flow or softening. The molecular chains penetrate into the crack fissures. The infiltrated polymer molecules then reconstruct a new network within the crack via the reformation of dynamic bonds (e.g., reformation of hydrogen bonds, disulfide bond exchange reactions), effectively bridging the fracture surfaces. This restores the structural continuity of the perovskite layer and reestablishes carrier transport pathways. As illustrated in Fig. 4a, the TA-NI SHP, employed as an additive within the photoactive layer, adheres to perovskite grain boundaries via coordination bonds [77]. Following crack formation induced by continuous bending, the TA-NI polymer, exhibiting room-temperature self-healing capability, repairs the grain boundary cracks at ambient conditions, reconstructing the inter-grain connections and restoring perovskite continuity. Quantitative studies reveal that after being bent 4000 times under a radius of 5 mm, the F-PSCs based on control thin films retained only 60% of their initial PCE, while devices modified with SHPs maintained 94% of the initial PCE. Notably, devices employing self-healing thin films all demonstrated impressive self-recovery capability after being stored at room temperature for 1 hour, with PCE recovering from 94% to 97%.

Beyond healing intralayer cracks at grain boundaries, SHPs applied via interfacial engineering can also repair bending-induced interfacial delamination. When deployed as an interfacial layer, polar groups (e.g., carboxyl, amino groups) in the SHPs form hydrogen bonds or ion–dipole interactions with chemical groups on the surfaces of the perovskite or charge transport layers (e.g., Pb–I bonds, –OH groups). Upon delamination caused by continuous bending, the SHP, triggered by a stimulus, softens and diffuses toward the delaminated interface. It wets the separated surfaces of the perovskite and the transport layer/electrode, effectively reconnect the interface. This action restores interfacial electrical contact, reduces interfacial resistance, and prevents crack propagation along the interface. As shown in Fig. 4b, the SHP poly(LA) functions as an interfacial layer between the perovskite and the HTL [78]. Its carboxylic acid groups interact with Pb^2+^ and I^−^ ions. After interfacial delamination induced by bending, self-healing is triggered at 65 °C, involving disulfide bond reconfiguration. This repairs the delamination, restores interfacial electrical contact, and reestablishes efficient carrier transport pathways across the interface. Quantitative studies demonstrate that during the continuous bending phase, the PCE of the control device declined to nearly 20% of its original efficiency after 3000 bending cycles, while the poly(LA)-modified device maintained > 80% of its initial PCE. After thermal annealing at 65 °C for 6 h, followed by storage in a nitrogen atmosphere at 25 °C, the efficiency could be restored to 95% of its initial value.

### Methods for Evaluating Perovskite Self-Healing Properties

Applying self-healing PSCs from conceptual demonstrations to practical technology requires the establishment of reliable and standardized evaluation approaches. Presently, cross-comparative analysis of healing efficacy across different studies is fundamentally constrained by the pervasive use of non-uniform damage induction methods and healing protocols. To address this pivotal challenge, this section proposes a foundational standardization paradigm and an idealized assessment workflow and also gives several multi-scale characterization techniques.

First, to establish a reproducible and comparable initial degraded state, the mandatory implementation of standardized aging protocols is paramount. We strongly advocate for the adoption of the International Summit on Organic PV Stability (ISOS) consensus protocols as the foundational benchmark. Pertinent tests include: dark storage test (ISOS-D), dark state bias stability test (ISOS-V), light stability test (ISOS-L), outdoor stability test (ISOS-O), thermal cycling test (ISOS-T), light-humidity-thermal cycling test (ISOS-LT), light–dark cycling test (ISOS-LC) and intrinsic stability test (ISOS-I) [79–81].

Next, the healing efficiency (*η*_heal_) must be quantified against a standardized damage benchmark and reported with complete procedural transparency. The core metric is defined as:

2 $$\eta_{{{\mathrm{heal}}}} = \left( {\frac{{{\mathrm{PCE}}_{{{\mathrm{healed}}}} }}{{{\mathrm{PCE}}_{{{\mathrm{initial}}}} }}} \right) \times 100\%$$

Critically, the reporting of *η*_heal_ is rendered meaningless without the concomitant specification of: The precise damage protocol employed (conforming to ISOS) the exact healing stimulus parameters (e.g., thermal annealing at 60 °C for 30 min under ambient atmosphere).

Besides, a singular healing event merely validates concept feasibility; practical utility mandates functional endurance. Consequently, evaluating healing durability via multiple consecutive “damage-healing” cycles is indispensable. The principal metric is the trajectory of *η*_heal_ as a function of cycle number (*n*). A stable or gradually declining *η*_heal_(*n*) signifies robust, persistent self-healing functionality. Furthermore, reporting critical stability parameters (e.g., *T*_80_, the duration for PCE to decay to 80% of its initial value) following successive healing cycles is of paramount importance.

**Fig. 5:**
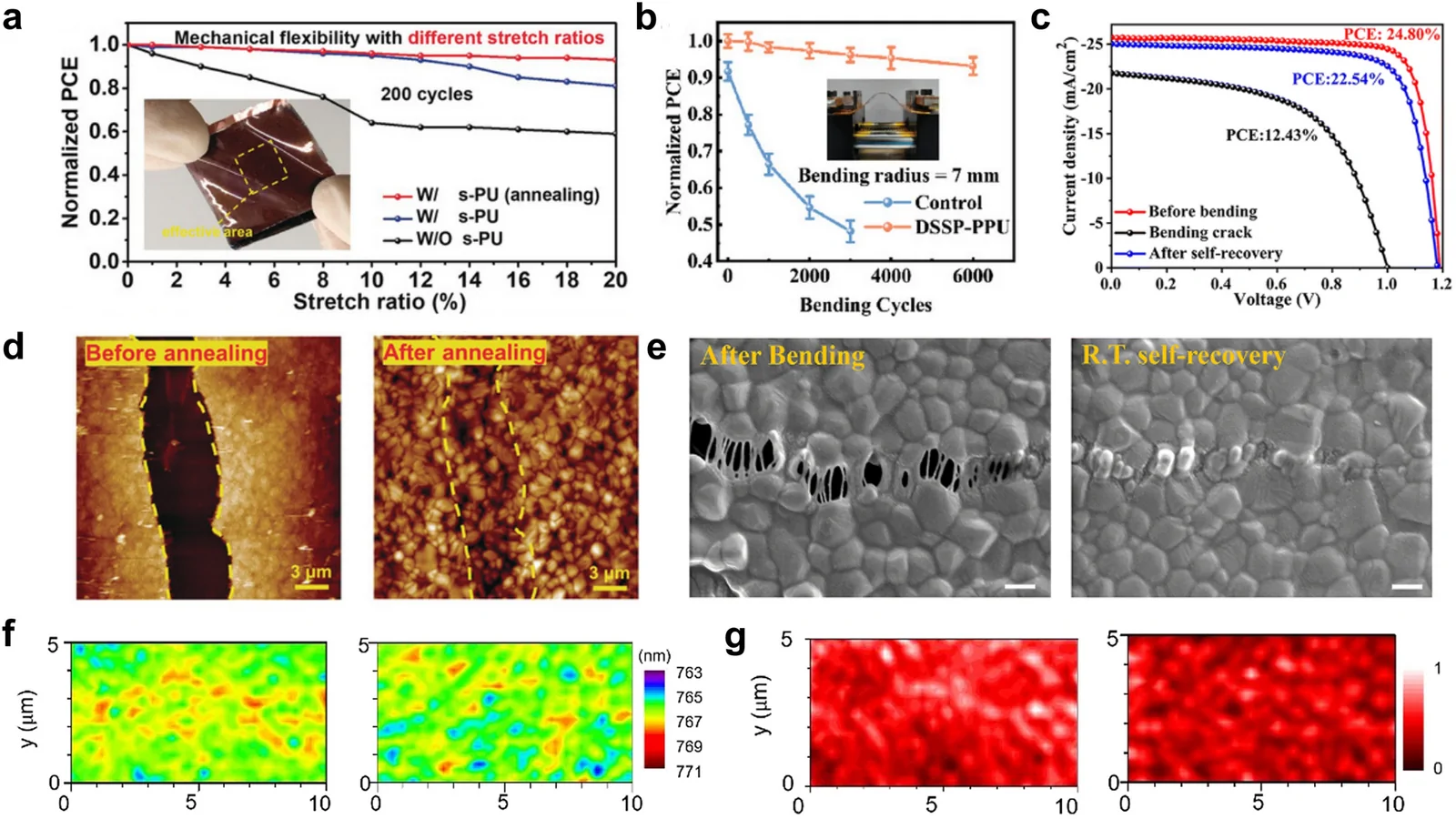
**a** Normalized average PCE of PSCs under different stretching forces for 200 cycles [82] Copyright 2020, Wiley–VCH; **b** PCE evolution of control and DSSP-PPU-doped F-PSCs under bending cycles [83] Copyright 2023, Wiley–VCH; **c** J-V curve [84] Copyright 2024, Royal Society of Chemistry; **d** In situ AFM images [82] Copyright 2020, Wiley–VCH; and **e** SEM images [84] Copyright 2024, Royal Society of Chemistry; Confocal photoluminescence maps of **f** peak wavelength and **g** peak intensity [85] Copyright 2022, Wiley–VCH

In addition, an ideal standardized evaluation workflow should be as follows: Induce reproducible damage using a benchmark aging protocol → characterize the damage state using multi‑scale methods (electrical, morphological, chemical) → trigger healing under well‑defined conditions → quantify the extent of recovery again using the same multi‑scale methods. Multi‑scale methods include:

For assessing electrical performance recovery, current density–voltage (J–V) characterization serves as the primary diagnostic tool. Systematic J–V measurements before degradation, after degradation, and after healing enable the calculation of *η*_heal_ and track the recovery of key photovoltaic parameters (*V*_OC_, *J*_SC_, FF) (Fig. 5a–c) [82–84]. Complementary techniques such as steady-state maximum power point tracking, external quantum efficiency measurements, and electrochemical impedance spectroscopy provide further insight into the restoration of interfacial contacts, bulk defect passivation, and ion migration suppression.

Microstructural and morphological recovery is commonly evaluated using scanning electron microscopy and atomic force microscopy to examine surface and cross-sectional features (e.g., grains, cracks, voids) (Fig. 5d, e) [82, 84]. Photoluminescence mapping via confocal fluorescence microscopy visualizes spatial variations in emission intensity, where PL recovery indicates reduced non-radiative recombination. This can be quantitatively supported by time-resolved photoluminescence to measure carrier lifetime restoration (Fig. 5f, g) [85].

Chemical and structural recovery is monitored through techniques such as X-ray diffraction (tracking phase changes, crystallinity, and strain), Fourier-transform infrared spectroscopy (probing dynamic bond reformation in polymers), and X-ray photoelectron spectroscopy (analyzing chemical states and degradation products).

For self-healing polymer encapsulants, the recovery of barrier functionality is critical for long-term device stability. Beyond basic hydrophobicity (e.g., water contact angle), the restoration of moisture and oxygen barrier properties must be quantified. Water vapor transmission rate (WVTR) and oxygen transmission rate (OTR) should be measured using standardized methods (e.g., ASTM F1249, ASTM D3985), with post-healing performance reported relative to the initial intact state to directly evaluate functional recovery [86, 87].

The trajectory toward credible, comparable, and ultimately translatable research on self-healing PSCs is predicated on the synergistic integration of a standardized assessment framework with multi-scale characterization. This section has delineated a coherent proposal encompassing unified damage/repair standards, a durability benchmark, and a rigorous sequential workflow that explicitly includes evaluating the functional recovery of encapsulation barriers. The widespread adoption of such an approach will transform key metrics—from the optoelectronic *η*_heal_ to the barrier WVTR_recovery_—from often ambiguous figures of merit into robust, universally comparable parameters. This paradigm shift is an essential prerequisite for the objective evaluation of novel material systems, the guidance of rational polymer design, and the conclusive demonstration of the operational longevity required for the commercialization of perovskite photovoltaics.

## Effects of SHPs in PSCs

**Fig. 6:**
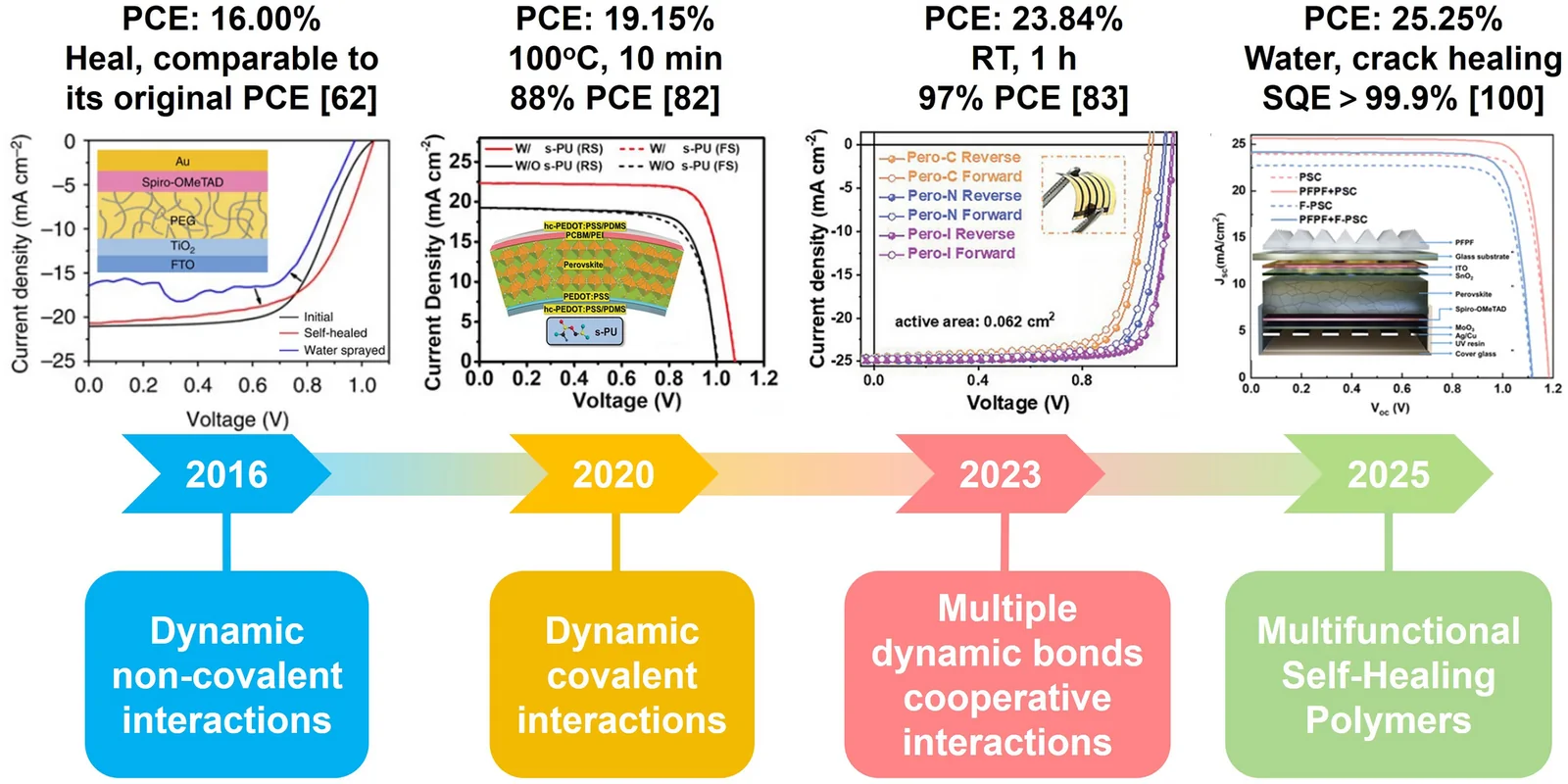
Development of SHPs-based PSCs (RT: room temperature; SQE: Pb^2+^ sequestration efficiency). [62] Copyright 2016, Springer Nature. [82] Copyright 2020, Wiley–VCH. [83] Copyright 2023, Wiley–VCH. [100] Copyright 2025, Royal Society of Chemistry

As a class of functional polymers, SHPs play multiple critical roles in PSCs. As illustrated in Fig. 6, the development of SHPs-based PSCs has evolved significantly. Initially, dynamic non-covalent interactions and dynamic covalent interactions functioned independently. Later, the introduction of multiple dynamic bonds working synergistically enhanced the self-healing efficiency and under milder conditions. Nowadays, the design of SHPs has become more functionalized, enabling additional functionalities to be integrated into PSCs. Emerging studies have demonstrated that SHPs enable dynamic defect passivation through continuous interactions between their functional groups (e.g., carboxyl and amino groups) and perovskite defect sites (e.g., undercoordinated Pb^2+^ ions and I^−^ vacancies), thereby regulating crystallization kinetics [88–90]. Furthermore, the elastic networks of SHPs can effectively buffer lattice strain within perovskite films, endowing the devices with superior mechanical flexibility [91]. More importantly, the intrinsic self-healing properties of SHPs provide an effective solution to mitigate performance degradation during long-term device operation. In this section, we comprehensively review the effects of SHPs in PSCs from three strategic perspectives: film additive engineering, interface engineering, and external encapsulation approaches.

### Effects of SHPs in Perovskite Films

**Fig. 7:**
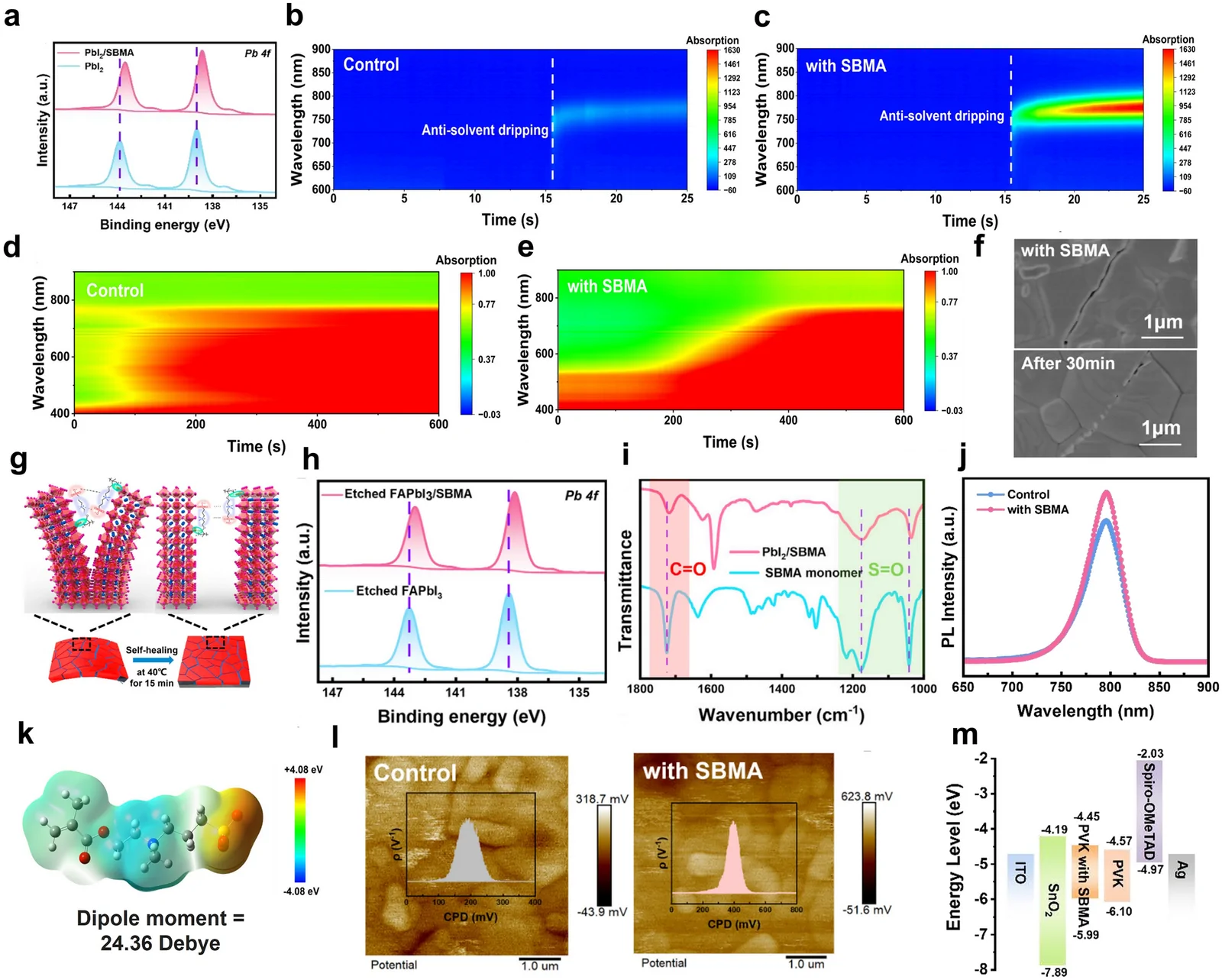
**a** Pb 4*f* for PbI_2_ and PbI_2_/SBMA film; Photoluminescence (PL) spectra of **b** pristine perovskite film and **c** perovskite film with SBMA during the spin-coating process. In situ UV–Vis absorption spectroscopy of **d** pristine perovskite film and **e** perovskite film with SBMA during the annealing process; **f** Top-view SEM images of ruptured and thermally treated perovskite films. **g** Self-healing process of perovskite film with SBMA; **h** High-resolution XPS spectra of Pb 4*f*; **i** FTIR spectra of pure SBMA and PbI_2_/SBMA; **j** Steady-state PL spectra; **k** Distribution of electrostatic potential (ESP) and dipole moments of SBMA calculated by density functional theory (DFT); **l** Topography KPFM images of pristine perovskite film and perovskite film with SBMA; and **m** Energy-level diagrams of PSCs [92] Copyright 2024, Elsevier

As functional additives, SHPs are introduced into the perovskite precursor solution system, offering an innovative strategy to address the inherent instability issues of perovskite films. SHPs not only confer self-healing capabilities to the perovskite but also regulate crystallization kinetics during film formation. The crystallization kinetics of perovskite films critically determine grain size, orientation, and defect density, serving as a core factor influencing device optoelectronic performance (e.g., carrier lifetime, open-circuit voltage) and stability. Perovskite precursor solutions undergo rapid crystallization during film processing (e.g., instantaneous nucleation induced by anti-solvent), often resulting in small grains, numerous grain boundaries, and pinhole defects, leading to severe non-radiative recombination and ion migration. The incorporation of SHPs modulates crystallization kinetics by retarding nucleation rates and guiding ordered grain growth, thereby yielding dense, large-grained perovskite films with reduced defects. Ge et al. synthesized 3-[[2-(methacryloyloxy)ethyl]dimethylammonio]propane-1-sulfonate (SBMA), which undergoes in situ cross-linking during perovskite film annealing [92]. The incorporation of the zwitterionic elastomer SBMA into the perovskite precursor solution orchestrates a multi-stage regulation of the crystallization process, ultimately leading to superior film quality. Initially, the polar functional groups of SBMA, namely the ester (C=O) and sulfonate (S=O), actively coordinate with the lead ions (Pb^2+^) from PbI_2_ in the precursor solution, as shown in Fig. 7a. This coordination alters the solvation chemistry, forming an intermediate SBMA-PbI_2_ adduct that modulates the precursor’s colloid properties and reaction kinetics. During the nucleation stage triggered by anti-solvent dripping, this preformed intermediate plays a pivotal role. It significantly lowers the energy barrier for the formation of the photoactive perovskite phase (α-FAPbI_3_). In situ photoluminescence studies (Fig. 7b, c) reveal a rapid and intense emission at ~ 780 nm, indicating immediate and dominant *α*-phase nucleation, effectively bypassing the formation of the undesirable non-perovskite *δ*-phase. This promotes a high density of homogeneous nuclei, ensuring complete surface coverage as described by the LaMer model [93]. Subsequently, in the crystal growth stage during thermal annealing, the SBMA-PbI_2_ adduct continues to exert influence. It retards the crystallization kinetics, as evidenced by in situ UV–Vis absorption spectroscopy, as shown in Fig. 7d, e. This delayed growth, consistent with the Ostwald ripening mechanism, provides sufficient time for mass transport and structural reorganization [94]. Consequently, the perovskite grains grow larger and more ordered, with fewer defects and pinholes, as the additive suppresses the detrimental dissolution–recrystallization processes often observed in control films. The regulated crystallization kinetics ultimately yielded large-grained perovskite films with self-healing functionality, as depicted in Fig. 7f. Following crack formation induced by 5000 bending cycles, the SBMA-containing perovskite film subjected to treatment at 40 °C for 30 min exhibited nearly complete crack healing.

Beyond modulating crystallization kinetics, the solution-processable nature and soft lattice characteristics of perovskites inherently lead to the formation of intrinsic defects (e.g., uncoordinated Pb^2+^, halide vacancies, grain boundary dangling bonds) during crystallization. These defects act as non-radiative recombination centers, significantly reducing carrier lifetime, triggering ion migration, and consequently degrading device efficiency and stability. SHPs, containing abundant functional groups (e.g., carboxyl, amino) within their molecular chains, enable dynamic defect passivation through sustained interactions with perovskite defect sites (e.g., uncoordinated Pb^2+^, I^−^ vacancies). This yields higher-quality perovskite films with substantially enhanced optoelectronic properties. A schematic illustrating the interaction between SBMA and the perovskite is presented in Fig. 7g. The chemical coordination of SBMA was directly confirmed by X-ray photoelectron spectroscopy (XPS) (Fig. 7h) and Fourier-transform infrared spectroscopy (FTIR) (Fig. 7i). The SBMA-incorporated film demonstrated a higher photoluminescence (PL) intensity (Fig. 7j), indicating effective passivation of trap states at the grain boundaries. Electrostatic potential (ESP) measurements (Fig. 7k) revealed a dipole moment of 24.36 Debye for SBMA, which is expected to establish a significant dielectric environment at the grain boundaries. Kelvin probe force microscopy (KPFM) measurements showed that the SBMA-treated perovskite film exhibited a substantially higher average contact potential difference (CPD) along with a narrower CPD distribution (Fig. 7l), confirming the formation of a homogenized dielectric environment. The increased average CPD directly verifies that SBMA molecules form a permanent dipole layer. This dipole layer effectively lowers the surface work function, inducing pronounced downward band bending at the perovskite surface and thereby optimizing the energy band structure, as illustrated in Fig. 7m.

**Fig. 8:**
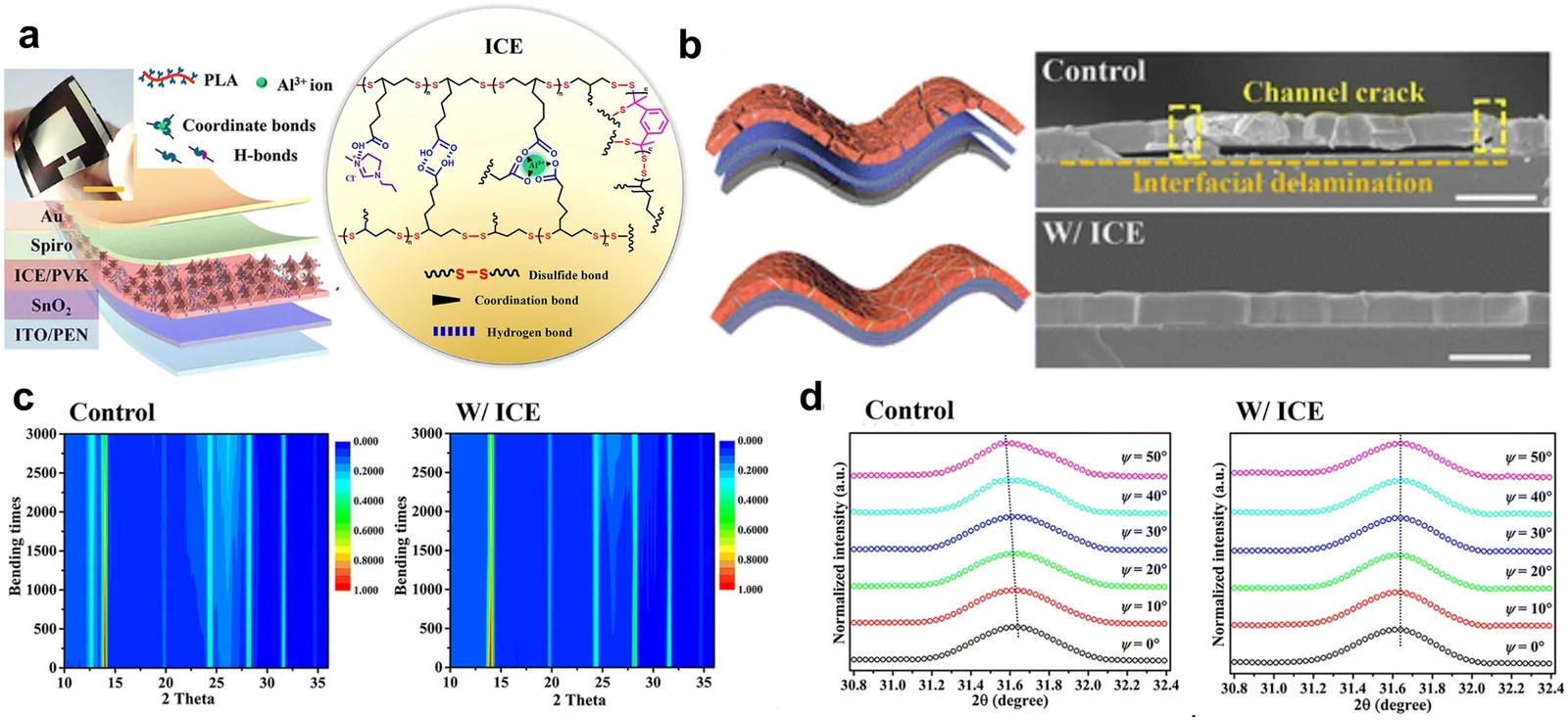
**a** Device configuration of flexible PSCs and cross-linked network structure of ICE; **b** Cross-sectional SEM images of the flexible PSCs tested for 5000 cycles; **c** In situ 2D X-ray diffraction (2D-XRD) of perovskite film under 3000 bending cycles; and **d** Grazing incidence X-ray diffraction (GIXRD) of perovskite film varies with ψ from 0° to 50° (φ = 0.5°) [84] Copyright 2024, Royal Society of Chemistry

Although perovskites exhibit exceptional optoelectronic properties for photovoltaics, their ionic crystal nature and inherent brittleness pose significant challenges for flexible applications. Perovskite films are prone to developing microcracks, grain boundary fractures, and interfacial delamination under repeated bending or mechanical stress, leading to hampered carrier transport, exacerbated non-radiative recombination, and rapid efficiency degradation. Xue et al. synthesized a self-healing ion-conducting elastomer (ICE) containing imidazolium-based ionic liquid as an additive incorporated into the perovskite precursor solution for fabricating f-PSCs, as shown in Fig. 8a [84]. Cross-sectional SEM images (Fig. 8b) after 5000 bending cycles (*R* = 3 mm) revealed distinct channel cracks and interfacial delamination in the control film, whereas the ICE-containing film maintained its structural integrity. In situ X-ray diffraction (XRD) peak monitoring during 3000 bending cycles (Fig. 8c) showed that the control film exhibited weakened characteristic peaks and a pronounced PEN substrate signal at ≈26°, resulting from perovskite grain detachment exposing the substrate. Conversely, the ICE-containing film showed negligible change in diffraction peak intensity after cycling. This stability is attributed to the cross-linked ICE network distributed along grain boundaries, protecting the brittle perovskite film. Additionally, grazing-incidence X-ray diffraction (GIXRD) analysis of lattice strain (Fig. 8d) showed that the scattering peak (~ 31.6°) of the control film shifted leftward as *ψ* increased from 0° to 50°, indicating the presence of tensile strain. This leftward shift was suppressed in the ICE-containing film, demonstrating effective mitigation of tensile strain. F-PSCs incorporating SHPs thus demonstrated significantly enhanced mechanical flexibility.

In summary, the additive engineering strategy employing SHPs leverages their intrinsic dynamic repair mechanisms and material chemistry to synergistically enhance multiple facets of the perovskite active layer, including microstructure, macroscopic mechanics, crystallization quality, defect state density, and interfacial energy-level alignment. This multifaceted functionality positions SHPs as a highly promising technological pathway for addressing the critical challenges of stability, efficiency, and flexibility in perovskite devices.

### Effects of SHPs in Perovskite Interfaces

The efficiency and stability of PSCs are intrinsically governed by charge carrier dynamics encompassing transport, extraction, collection, and recombination processes with the bulk of the perovskite films and at the interfaces. Taking n-i-p structured PSCs as an example, transparent conducting oxide (TCO)/ETL/perovskite/HTL/electrode configuration gives four critical interfaces determining device performance. Solution-processed crystallization and thermal annealing inevitably induce deep-level defects at perovskite surfaces, exacerbating interfacial recombination loss [78]. Consequently, perovskite-related interfaces (ETL/perovskite and perovskite/HTL) represent focal points for optimization strategies. The incorporation of SHPs has established a novel paradigm for dynamic interfacial engineering, integrating mechanical compliance and self-repair functionality at perovskite grain boundaries.

**Fig. 9:**
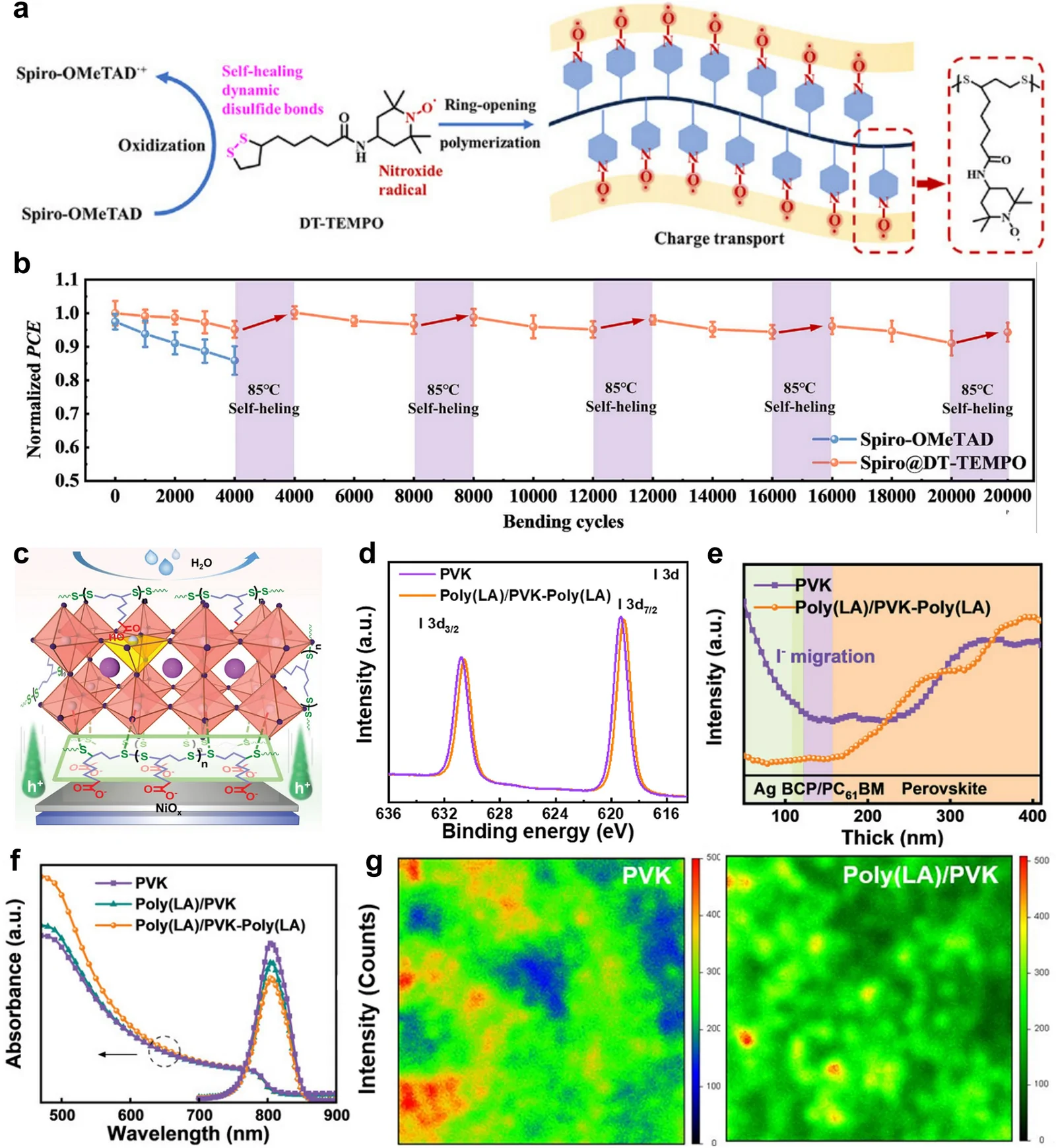
**a** Schematic illustration of DT-TEMPO cross-linking polymerization and p-doping in Spiro-OMeTAD; **b** Mechanical stability test of unencapsulated F-PSCs [95] Copyright 2025, Wiley–VCH; **c** Schematic illustration of self-healing in interface; **d** XPS spectra of I 3*d*; **e** Linear energy-dispersive spectrometer elemental mapping profiling of the device section; **f** UV–Vis absorption spectra and steady-state photoluminescence (PL) spectra; and **g** 2D photoluminescence images [78] Copyright 2024, Wiley–VCH

Recent years have witnessed significant advancements in the application of SHPs for interfacial engineering in PSCs. Gao et al. proposed an innovative strategy that integrates a self-healing polymer into the hole-transport layer [95]. They achieved this by doping Spiro-OMeTAD with a novel multifunctional self-healing nitroxide radical monomer, 4-[[5-(1,2-dithiolane-3-yl)-1-oxopentyl]amino]-2,2,6,6-tetramethylpiperidin-1-oxyl (DT-TEMPO). The incorporation of dynamic disulfide bonds, capable of cleavage and reformation, endowed Spiro-OMeTAD with a self-healing capability. When external thermal annealing was applied at temperatures exceeding the critical threshold (76 °C) for the cleavage and reformation of the disulfide bonds, the disulfide-containing five-membered ring was activated, initiating a ring-opening polymerization. This process generated sulfur radicals, which could accept electrons and be reduced to DT-TEMPO anions, while Spiro-OMeTAD lost electrons and was oxidized to form Spiro-OMeTAD•^+^, as illustrated in Fig. 9a. This mechanism enhanced the oxidation rate of Spiro-OMeTAD. Furthermore, scratches on the poly(DT-TEMPO) film formed after ring-opening polymerization could self-heal within 5 min at 85 °C. Devices employing Spiro@DT-TEMPO exhibited nearly complete recovery of their PCE after only 10 min of thermal annealing at 85 °C, as shown in Fig. 9b. Remarkably, the efficiency maintained approximately 95% of its initial value after multiple healing cycles, demonstrating a “self-healing cycle”. The underlying dynamic chemical repair mechanism shares significant mechanistic similarity with that revealed in recent studies on SHPs incorporated into the perovskite bulk [62–64].

SHPs incorporated into the interfacial layer not only confer self-repairing functionality to the perovskite but have also been demonstrated in related studies to passivate surface defects, enhance the uniformity of the perovskite film surface, and simultaneously suppress ion migration. For instance, Chen et al. introduced *α*-lipoic acid (LA) into the interface between the interfacial layer and the perovskite layer [78]. As illustrated in Fig. 9c, ring-opening polymerization of LA within the interfacial layer formed a multifunctional cross-linked network comprising dynamic covalent disulfide bonds and non-covalent carboxyl hydrogen bonds (–COOH). As shown in Fig. 9d, the carboxylic acid groups formed hydrogen-bonding interactions with iodide ions (–OH…I). Since grain boundaries (GBs) serve as the primary pathways for ion transport within perovskite films, the poly(LA) interfacial layer acts as a barrier layer. It suppresses the migration of I^−^ ions through these hydrogen-bonding interactions. Linear energy-dispersive spectrometer was employed to probe the extent of internal migration and degradation within light-aged devices (Fig. 9e). Severe iodine accumulation was observed within the electrodes of conventional PVK-based photovoltaic cells. Notably, devices assembled using the poly(LA)/PVK-poly(LA) configuration exhibited an extremely low interfacial I^−^ ion concentration, confirming effective ion migration suppression. Furthermore, steady-state photoluminescence (PL) spectroscopy verified the passivation of surface defects, leading to improved carrier transport (Fig. 9f). Time-resolved confocal fluorescence imaging further indicated that poly(LA)/PVK exhibited reduced fluorescence intensity and a more uniform distribution of fluorescence emission as compared to pristine PVK (Fig. 9g).

### Effects of SHPs for Encapsulation

Encapsulation constitutes an indispensable step in the commercialization trajectory of PSCs. The pronounced sensitivity of perovskite materials to humidity, oxygen, UV irradiation, and thermal stress triggers deleterious phenomena including ion migration, phase segregation, and chemical decomposition, critically undermining long-term operational stability [96]. Technically, PSC encapsulation refers to the process of safeguarding cell units/modules through material-based and engineering interventions, which effectively isolates the active layers from moisture ingress, oxidative degradation, and environmental corrosives [97]. Under outdoor operational conditions, PSC modules inevitably sustain mechanical damage from rain, snow, hail, or fire events. Compromised encapsulation layers permit toxic lead ion leaching from damaged regions during precipitation, posing substantial risks of environmental contamination. Manual restoration of degraded photovoltaic modules remains impractical due to intricate structural integration. Consequently, incorporating SHPs into encapsulation architectures delivers dual functionality: (1) autonomously restoring barrier integrity to enhance device durability and (2) immobilizing lead species via dynamic bond reconfiguration to prevent eco-toxic leakage.

**Fig. 10:**
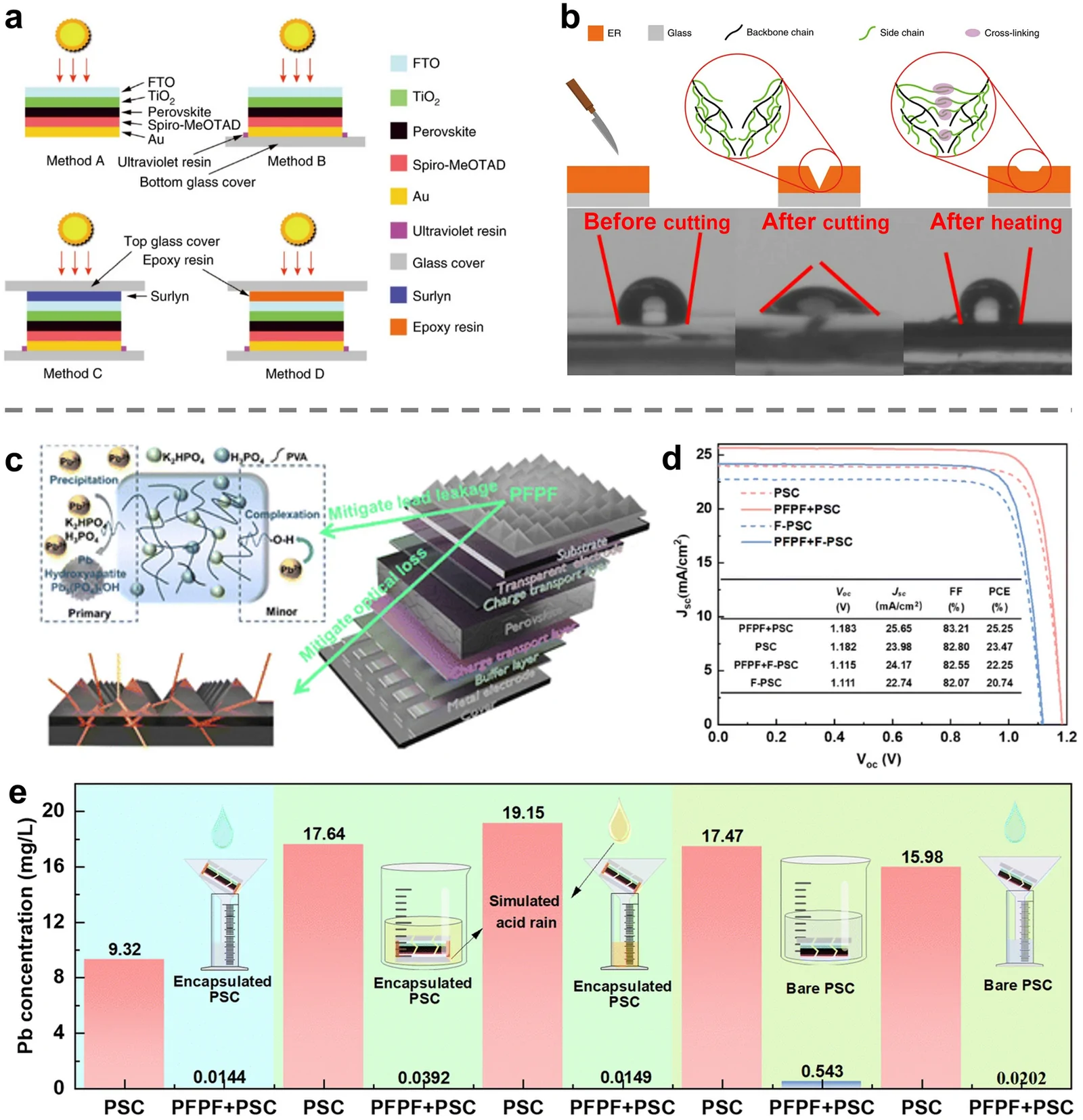
**a** Schematic showing the encapsulation methods A, B, C, and D; **b** Schematic showing the self-healing process of the ER encapsulant and the photos show the water contact angle measurement on the film [98] Copyright 2019, Springer Nature; **c** Illustration of encapsulation architectures of PFPFs; **d** J-V curves of PSCs; and **e** summary of equilibrium Pb^2+^ concentrations for different tests [100] Copyright 2025, Royal Society of Chemistry

Qi et al. pioneered the application of self-healing epoxy resin as an encapsulation material for PSCs, systematically evaluating three distinct encapsulation methodologies as illustrated in Fig. 10a [98]. Their work established foundational guidelines for subsequent research on self-healing encapsulation systems. The healing efficacy was quantitatively assessed through water contact angle measurements before and after damage-healing cycles. Experimental results revealed a significant reduction in contact angle (indicating compromised hydrophobicity) in damaged encapsulation layers, which directly correlated with diminished moisture and oxygen barrier performance. Remarkably, upon sunlight-induced thermal treatment, the water contact angle recovered to near its initial value, demonstrating restored hydrophobic properties (Fig. 10b). This study conclusively demonstrates that SHPs can autonomously repair encapsulation layer damage, thereby providing durable hydrophobic protection for PSCs.

However, the curing of epoxy resins usually requires high-temperature curing, which may have adverse effects on the device. Zhong et al. engineered a self-healing polyurethane adhesive (PUA) to enable both lead precipitation and facile encapsulation of attachable PSCs [99]. Building upon these foundational works, Jiang et al. synthesized phosphate-buffered functionalized polymer films (PFPFs) with intrinsic self-healing properties [100]. The PFPFs demonstrate robust adhesion to glass substrates through hydrogen-bonding interactions with surface Si–OH groups. This enabled the fabrication of pyramid-structured PFPF films via a casting process, which were subsequently laminated onto the light-incident side of PSC devices (Fig. 10c). The pyramidal architecture of PFPFs confers exceptional anti-scattering characteristics, enhancing light transmittance and consequently improving device efficiency from 23.47% to 25.25% (Fig. 10d). More significantly, PFPF-encapsulated devices exhibit remarkable environmental resilience, effectively preventing lead leakage under various harsh conditions (Fig. 10e). This breakthrough represents a paradigm shift beyond conventional encapsulation strategies that merely provide physical protection. This technology utilizes a pyramidal architecture for light management, offering exceptional environmental barrier properties and a distinctive capability for underwater self-healing at room temperature. Through simulated hail impact—achieved by dropping a 45 mm diameter, 360 g metal ball onto the ITO glass side to fracture the encapsulated PSCs—followed by exposure to an acidic rain environment (25 °C, RH = 100%, pH = 4.2), the encapsulation layer autonomously repairs damage within minutes. This self-healing function enables the restoration of encapsulation integrity during outdoor operation, while also significantly enhancing overall device performance.

In summary, the stability of PSCs is significantly enhanced by self-healing polymer encapsulation. Even if the encapsulation layer is damaged, it can maintain its barrier function against moisture and oxygen ingress while effectively immobilizing lead ions to prevent leakage. This dual functionality is crucial for ensuring both the long-term performance of PSCs and their environmental safety.

### Major Concerns of SHPs in PSCs

Although SHPs have been shown to improve photoelectric properties and mechanical stability, several major concerns that may limit device performance must be acknowledged.

1. * Long-Term Aging and Chemical Stability Risks*:
  during service, polymeric materials undergo a series of changes in chemical composition and structure due to the combined effects of environmental factors such as heat, oxygen, moisture, light, microorganisms, and chemical media [101]. This degradation, termed “aging,” leads to deterioration in physical properties, including hardening, stickiness, embrittlement, discoloration, and loss of strength. The aging of SHPs and its negative impact on PSCs cannot be overlooked. However, research within the field of self-healing PSCs has rarely addressed this aspect, resulting in insufficient understanding of how SHP aging specifically affects perovskites. Potential negative impacts may include:
  Aging of SHPs is accompanied by decay in self-healing capability and the generation of degradation by-products. Reduced self-healing efficacy may leave subsequent damage to the perovskite layer unrepaired, allowing these defects to propagate and ultimately causing device failure. Degradation by-products from aged SHPs may directly damage the perovskite. For instance, under photochemical aging mechanisms, covalent bond cleavage in polymers exposed to UV light can generate alkoxy radicals (RO·), which may attack the perovskite crystal structure and accelerate the formation of metallic lead (Pb^0^) [102]. Some SHPs undergo yellowing and become opaque upon aging, potentially interfering with light absorption by the perovskite layer. The specific impacts of SHPs aging on perovskite stability and performance warrant thorough investigation.

(2) *Insulating Nature and Charge Transport Obstruction*:
  the inherent insulating nature of SHPs can hinder charge transport in PSCs. Most SHPs (e.g., polyurethanes, polydimethylsiloxane) possess wide bandgaps (> 5 eV), forming physical barriers at perovskite grain boundaries or interfaces. This impedes the cross-interface transport of charge carriers (electrons/holes). Mitigation strategies include the strategic design of conductive SHPs (e.g., by incorporating π-conjugated units or doping with conductive nanoparticles) or confining them to grain boundary regions that are not primary charge transport pathways (e.g., via vertically oriented self-assembly).

(3) *Thermal Expansion Mismatch and Interfacial Delamination*:
  SHPs can undergo volumetric changes in response to temperature fluctuations. This occurs because increased thermal energy intensifies the motion of polymer chains, leading to an increase in the average inter-chain distance and consequent material expansion. A critical concern for PSCs arises from the significant difference in the coefficients of thermal expansion (CTE) between SHPs and the perovskite material. Upon heating, this CTE mismatch can induce interfacial stress, ultimately leading to delamination at the SHPs/perovskite interface. Introducing strong anchoring groups is one of the solutions.

## Practical Approaches of Applying SHPs in PSCs

The potential benefits of integrating SHPs into PSCs are extensive. However, a major challenge lies in effectively incorporating these polymers into the device architecture. This section summarizes practical integration approaches, including ex situ and in situ methods. It also highlights several representative classes of SHPs, such as polyurethanes (PUs), polydimethylsiloxane (PDMS), addition polymers, ring-opening polymers, and copolymers. Currently, most SHPs applied in PSCs are introduced via ex situ polymerization and incorporated into perovskite films as functional additives to impart self-healing capability. As highly efficient additives, SHPs can significantly enhance the performance and stability of devices. Their design principles should be functionality-oriented, requiring the polymer to possess: 1) flexible chain segments to enhance perovskite flexibility, 2) electron-rich groups for defect passivation, crystallization regulation, and carrier transport optimization, and 3) dynamic covalent or non-covalent bonding groups to endow self-healing capabilities. Most of the molecules are self-healing polyurethane.

### Polymerization Methods of Introducing SHPs into PSCs

#### Ex Situ ***Polymerization***

**Fig. 11:**
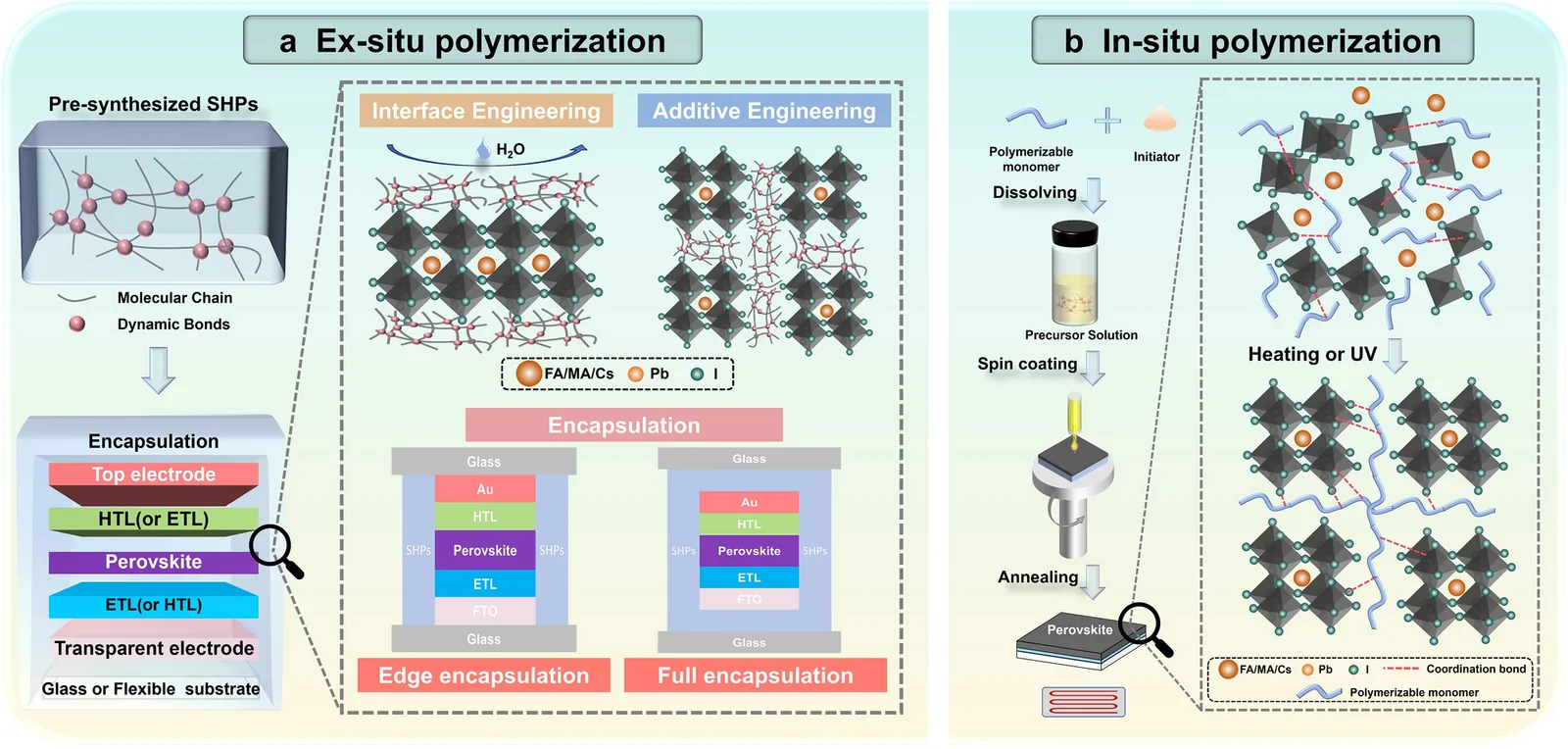
SHPs introducing strategy **a** ex situ polymerization; **b** in situ polymerization

Pre-synthesized SHPs can be feasibly applied to fabricate self-healing PSCs, and an ex situ polymerization is used to name this type of methods [103]. Pre-synthesized polymers exhibit well-defined properties with minimized batch-to-batch variability, enhanced controllability, enabling precise pre-design of polymer parameters (e.g., molecular weight, functional groups) and customization of passivation moieties, rendering them suitable for scalable manufacturing. This approach integrates polymers into PSCs architectures via solution processing or direct blending to achieve self-restorative functionality. Relevant studies have introduced SHPs into perovskite grain boundaries, interface layers, and encapsulation through ex situ polymerization, which has been verified to significantly improve the performance and stability [104]. To better illustrate this ex situ polymerization approach, Fig. 11a schematically illustrates the incorporation of SHPs into perovskite grain boundaries, interfacial layers, and encapsulation structures via ex situ polymerization techniques. These pre-synthesized SHPs typically contain dynamic bonds (e.g., disulfide, hydrogen bond) and passivating functional groups, enabling simultaneous self-healing capability and defect passivation. Importantly, they exhibit sufficient solubility in polar aprotic solvents (e.g., DMF, DMSO) commonly used in perovskite processing. Among these, polyurethane-based SHPs represent the most extensively studied category for ex situ polymerization, demonstrating remarkable achievements in both device efficiency enhancement and autonomous repair performance.

However, this strategy faces inherent limitations: Interfacial compatibility is a major challenge. The pre-synthesized polymers must be carefully designed for solubility in processing solvents to prevent phase separation. Even when blended, achieving molecular-level intimacy at the perovskite grain boundaries or device interfaces is difficult. Poor compatibility can lead to non-uniform coverage, weak adhesion, and the formation of voids or insulating layers that hinder charge transport and reduce mechanical integrity [105]. And, the introduction of preformed, often high-molecular-weight polymers can significantly interfere with crystallization kinetics. These polymers may act as physical barriers, impeding ion diffusion and mass transport during annealing. This can lead to suppressed grain growth, increased nucleation density, and potentially more grain boundaries [106]. While sometimes used as templating agents, the primary kinetic role is often passive obstruction, requiring precise optimization of polymer loading to avoid detrimental effects on film morphology. Notably, most SHPs exhibit insulating characteristics, where excessive incorporation may impede charge carrier transport or compromise perovskite crystallization quality, ultimately degrading device performance. These trade-offs necessitate stringent optimization of polymer loading to balance self-healing efficacy and photovoltaic metrics, particularly PCE.

#### In Situ ***Polymerization***

In situ polymerization refers to the preparation of self-healing polymer-modified perovskite devices using polymers indirectly obtained from polymerizable monomers under external stimuli (e.g., thermal, UV, moisture initiation, or initiator-triggered methods) [107], as shown in Fig. 11b. This approach circumvents the issues of poor solubility and non-uniform distribution in perovskite films associated with ex situ polymerization polymers. This method inherently promotes superior interfacial compatibility and uniformity. The monomers permeate the precursor solution, and the polymerization reaction occurs within the forming perovskite film, leading to the creation of an intertwined, conformal polymer network. This results in a more seamless and intimate interface at the molecular level, with the polymer matrix embedded within grain boundaries and at device interfaces. This enhanced compatibility improves interfacial adhesion, passivation, and stress distribution, contributing to both mechanical and environmental stability [108–110].

Moreover, the excellent solubility of polymerizable monomers ensures their homogeneous dispersion in the perovskite precursor solution. This uniformity allows early-stage interaction with precursors, thereby influencing key stages of crystallization. Initially, the monomers participate in solvent–solute coordination, which can modulate the formation and stability of critical intermediate phases such as Lewis acid–base adducts [111]. During annealing, polymerization is triggered, forming a uniform polymer network throughout the perovskite film. This integrated network actively regulates crystallization kinetics: During nucleation, monomers and the growing polymer establish binding interactions (e.g., hydrogen bonding or coordination) with precursors, reducing the effective concentration of free species, lowering the crystallization driving force, and delaying nucleation. This results in fewer, more controlled nucleation sites [112]. Concurrently, evolving linear or branched polymer chains physically restricts ion diffusion, further suppressing secondary nucleation. In the grain growth stage, the homogeneous polymer network acts as a confining framework that guides oriented crystal growth by spatially directing mass transport and limiting random growth directions [113]. Together—by modulating nucleation–growth kinetics, influencing precursor coordination, and providing a guiding scaffold—this approach promotes the formation of high-quality, large-grained, highly crystalline perovskite films. Such multifaceted control makes the in situ polymerization method particularly suited for precursor solution processing.

While in situ polymerization is conceptually attractive for integrating SHPs into PSCs, its practical implementation is fraught with challenges. These include the high risk of perovskite damage during processing (thermal initiation can lead to thermal runaway, causing thermal degradation of the perovskite, while photoinitiation can induce photodegradation of the perovskite), difficulties in achieving uniform and complete polymerization without detrimental shrinkage, the persistence of harmful residues, and significant hurdles in process control and scalability. Careful monomer/initiator selection, meticulous process optimization, and strategies to mitigate these specific defects are crucial for advancing this approach. However, this does not imply methodological limitations. Although fewer reports exist in this specific domain, polymer PSCs field research has already demonstrated the feasibility of applying in situ polymerization to charge transport layers and encapsulation layers. Developing highly conductive self-healing polymer interfacial layers or superhydrophobic self-healing encapsulation layers through in situ polymerization represents a critical need for advancing self-healing PSC technology.

### Representative SHPs in Self-Healing PSCs

#### Synthesis Strategies of SHPs Based on Step-Growth Polymers

In step-growth polymers, PUs are promising candidates to address the performance and stability challenges of perovskite materials. PUs are a class of versatile polymeric material synthesized via step-growth polymerization between isocyanates (R–NCO) and polyols, characterized by carbamate groups (–NHCOO–) in their backbone [114]. By adjusting monomer types (e.g., diisocyanates, polyols, chain extenders) and synthesis protocols, the chemical structure, mechanical properties, and functional characteristics of PUs can be highly tailored. Functional groups (e.g., carboxyl, amino, or dynamic bonds) can be introduced through chemical modifications, enabling tunable mechanical properties ranging from flexible elastomers to rigid plastics, alongside electrical insulation, weather resistance, and interfacial compatibility [115]. Furthermore, the microphase-separated structure of PU soft and hard segments endows it with both high elasticity and fatigue resistance, effectively buffering external stresses (e.g., bending, thermal expansion) and mitigating mechanical damage in brittle materials (e.g., perovskite films). When integrated with dynamic covalent bonds (e.g., disulfide bonds, Diels–Alder adducts) or supramolecular interactions (e.g., hydrogen bonds, metal coordination), PU gains unique functionalities such as self-healing, shape memory, and stimuli responsiveness.

**Fig. 12:**
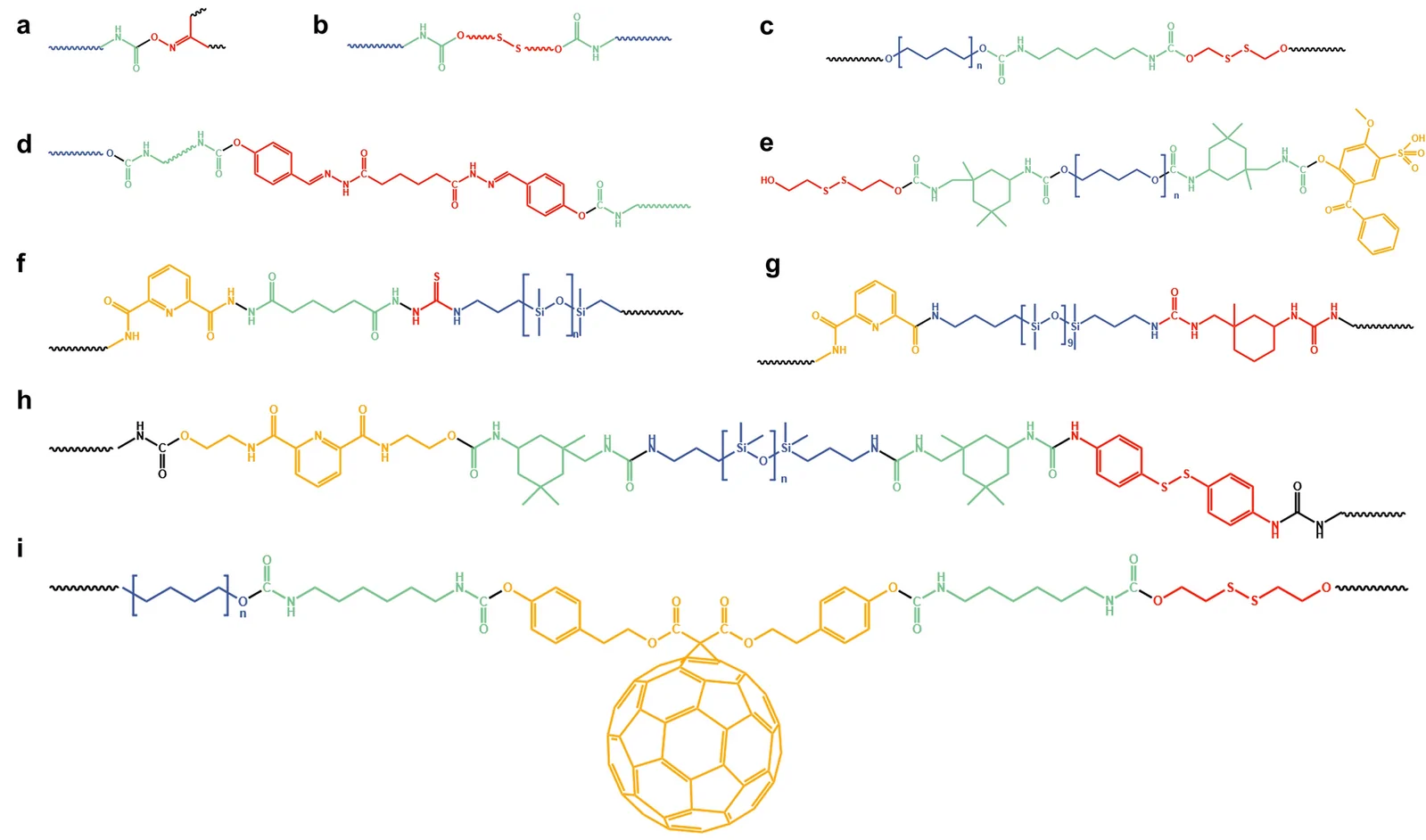
Chemical structure of representative SHPs based on PU **a** s-PU [82]; **b** PUDS [116]; **c** Self-healing PU [117]; **d** Ab-WPU [118]; **e** PUA [99]; **f** PAT [91]; **g** SHP [89]; **h** DSSP-PPU [83]; and **i** C60-PU [90]

As depicted in Fig. 12 and Table 1, representative chemical structures of self-healing polyurethanes (self-healing PUs) applied in PSCs in recent years are illustrated. The core molecular structure is using soft segments, hard segments, chain extenders, and dynamic bonds. The main difference between these typical structures lies in chain extenders and dynamic bonds. Chain extenders serve as the key component for introducing electron-rich functional groups, while dynamic bonds serve as the component for introducing self-healing performance. Meng et al. pioneered the incorporation of self-healing polyurethane (s-PU) into F-PSCs [82]. The s-PU was synthesized via polymerization of poly(tetramethylene ether glycol) (PTMEG) with multifunctional oximes and hexamethylene diisocyanate at room temperature. When introduced into F-PSCs as an additive through ex situ polymerization, the s-PU scaffold enabled the device to recover 93% of its initial efficiency after annealing at 100 °C for 10 min, establishing a robust methodology for integrating self-healing PUs. However, a healing temperature of 100 °C carries the risk of thermal decomposition for perovskite materials such as MAPbI_3_, while FAPbI_3_ may undergo a phase transformation under such conditions [70, 78]. Therefore, mild self-healing conditions have become the focus of subsequent research. In order to functional properly in PSCs applications, these self-healing PUs exhibit a universal structural design principle as follows:

1. Soft segments for elasticity: All self-healing PUs incorporate flexible soft segments (e.g., polyether, polyester or polysiloxane chains), which confer high elasticity to the polymer matrix, thereby enhancing the mechanical compliance of perovskite films under bending stress. Compared with polyether and polyester, polysiloxane showed better hydrophobicity and weather resistance.
2. Hard segments via isocyanate modulation: Isocyanates are universally employed as rigid segments. The high reactivity of –NCO groups provides active sites for functional group grafting (e.g., dynamic bonds or electron-rich moieties).
3. Electron-rich functional groups: Structures consistently integrate electron-donating groups such as carbonyl (–C=O), amino (–NH_2_), or phosphonic acid moieties. These groups form strong coordination interactions with undercoordinated Pb^2+^ ions and I⁻ vacancies on perovskite surfaces, effectively suppressing defect density and non-radiative recombination.
4. Dynamic bonds for self-healing: All designs incorporate dynamic covalent bonds (e.g., disulfide, Diels–Alder, or boronic ester linkages) or reversible non-covalent interactions. These bonds enable dynamic bond exchange, facilitating closed-loop repair of grain boundary cracks under mild stimuli (e.g., heat, light, or moisture).

**Table 1:** Self-healing performance and photovoltaic parameters of representative SHPs based on PU

Self-healing polymer	Introducing strategy	SHPs action site	Dynamic bonds	Functional Group	Self-healing conditions	Self-healing efficiency (%)	Device structure	PCE (%)	Stability	Refs
s-PU	Ex	Additive engineering	Dynamic oxime	Carbonyl Group	100 °C, 10 min	88	PDMS/hc-PEDOT:PSS/Perovskite(s-PU)/PEDOT:PSS/PEI/hc-PEDOT: PSS	19.15	Air 3000 h 90%PCE_initial_	[82]
PUDS	Ex	Additive engineering	Disulfide bond	Carbonyl Group	80 °C, 10 min	88	PEN/ITO/NiO_x_/Perovskite(PUDS)/PC_61_BM/Ag/PEI	20.30	25 °C 20%RH 3000 h 95%PCE_initial_	[116]
Self-healing PU	Ex	Additive engineering	Disulfide bond	Carbonyl Group	80 °C	90	FTO/c-TiO_2_/Perovskite(PU)/Carbon	10.61	85 °C 20%RH 800 h 102% PCE_initial_	[117]
C_60_-PU	Ex	Additive engineering	Disulfide bond	Fullerene Group	80 °C, 15 min	78	FTO/SnO_2_/Perovskite(C_60_-PU)/Spiro-OMeTAD/Ag	21.36	25 °C 40%RH 500 h 60% PCE_initial_	[90]
PUA	Ex	Encapsulation	Disulfide bond, Hydrogen bond	Sulfonic Acid Group	RT, 45 min	100	FTO/ETL/Perovskite/Spiro-OMeTAD/AU/PUA/Glass	24.15	65 °C 55%RH 2000 h 92% PCE_initial_	[99]
Ab-WPU	Ex	Additive engineering	Hydrogen bond, Acylhydrazone bond	Carboxyl and Carbonyl Group	60 °C, 30 min	95	PEI/MeO-2PACz/Perovskite(Ab-WPU)/PC_61_BM/BCP/Ag	21.27	25 °C N_2_ 2800 h 90%PCE_initial_	[118]
SHP	Ex	Additive engineering	Hydrogen bond	Pyridyl and Carbonyl Group	RT, 2 h	80	PDMS-SS-IP/hc-PEDOT:PSS/Perovskite(SHP)/PCBM/hc-PEDOT:PSS/PDMS-SS-IP	19.50	Air 3000 h 80% PCE_initial_	[89]
PAT	Ex	Additive engineering	Thiourea hydrogen bonds	Pyridyl and Carbonyl Group	100 °C, 15 min	85	PDMS/hc-PEDOT:PSS/Perovskite(PAT)/PEDOT:PSS/PEI/Ag	19.58	25 °C 20%RH 2000 h 83% PCE_initial_	[91]
DSSP-PPU	Ex	Additive engineering	Disulfide bond, Hydrogen bond	Pyridyl and Carbonyl Group	RT, 1 h	97	PEN/ITO/SnO_2_/Perovskite(PSSP-PPU)/Spiro-OMeTAD/Ag	22.24	20–30%RH 1300 h 100% PCE_initial_	[83]
PAB	Ex	Additive engineering	Disulfide bond, Hydrogen bond	Pyridyl and Carbonyl Group	Air, 2 h	–	PEN/FTO/SnO_2_/Perovskite(PAB)/Spiro-OMeTAD/Au	21.02	25 °C 35%RH 2000 h 80% PCE_initial_	[25]
s-PU	In	Additive engineering	Disulfide bond	Carbonyl Group	80 °C, 5 min	91	FTO/c-TiO2/m-TiO_2_/Perovskite(s-PU)/Carbon	17.42	25 °C 50%RH 408 h 79% PCE_initial_	[119]

#### Synthesis Strategies of SHPs Based on Addition Polymers or Open Ring Polymers

SHPs based on double bond radical polymerization and α-lipoic acid (LA) ring-opening polymerization represent promising candidates for in situ polymerization and are widely adopted synthesis strategies in PSCs, as depicted in Table 2.

**Table 2:** Self-healing performance and photovoltaic parameters of representative SHPs based on thioctic acid or double bond

Self-healing polymer	Introducing strategy	SHPs action site	Dynamic bonds	Functional Group	Self-healing conditions	Self-healing efficiency (%)	Device structure	PCE (%)	Stability	Refs.
Poly(TA)	In	Interface and additive engineering	Disulfide bond	Carboxyl Group	Dark storage	98	FTO/TiO_2_/TiO_2_-Poly(TA)/Perovskite(Poly(TA))/Spiro-OMeTAD/Au	20.4	25 °C 40%RH 1440 h 93% PCE_initial_	[122]
ICE	Ex	Additive engineering	Disulfide bond, Hydrogen bond, Coordination bond	Imidazolyl and Carboxyl Group	RT, 1 h	91	PEN/ITO/SnO_2_/Perovskite(ICE)/Spiro-OMeTAD/Au	24.84	25 °C N_2_ 5000 h 90% PCE_initial_	[84]
TA-NI	In	Additive engineering	Disulfide bond, Hydrogen bond	Ammonium Group	RT, 1 h	97	PEN/ITO/SnO_2_/Perovskite(TA-NI)/Spiro-OMeTAD/Au	23.84	30%RH Air 3000 h 93% PCE_initial_	[77]
Poly(LA)	In	Interface and additive engineering	Disulfide bond, Hydrogen bond	Carboxyl Group	65 °**C**, 6 h	95	PET/ITO/NiO_x_/Poly(LA)/Perovskite-Poly(LA)/PC_61_BM/BCP/Ag	19.03	25 °C 50%RH 2000 h 80% PCE_initial_	[78]
SBMA	In	Additive engineering	Electrostatic bonding	Carbonyl and Sulfonic Acid Group	40 °C, 15 min	98	PEN/ITO/SnO_2_/Perovskite(SBMA)/Spiro-OMeTAD/Ag	24.51	ISOS-L-1 500 h 90% PCE_initial_	[92]
ADP	In	Additive engineering	Hydrogen bond, Electrostatic interaction	Amino and Carbonyl Group	60 °C, 30 min	90	PEN/ITO/SnO_2_/Perovskite(ADP)/Spiro-OMeTAD/Ag	23.16	30%RH Air 1300 h 91% PCE_initial_	[123]

The mechanism of double bond radical polymerization comprises three stages: chain initiation, chain propagation, and chain termination. Initiators (e.g., photoinitiators or thermal initiators) decompose under external stimuli (light or heat), generating active free radicals (R·). These radicals attack monomers containing double bonds (e.g., acrylates), cleaving the double bonds to form new radical chains. These chains continuously incorporate monomers, leading to chain elongation. The reaction terminates via radical coupling (combination) or chain transfer [120].

**Fig. 13:**
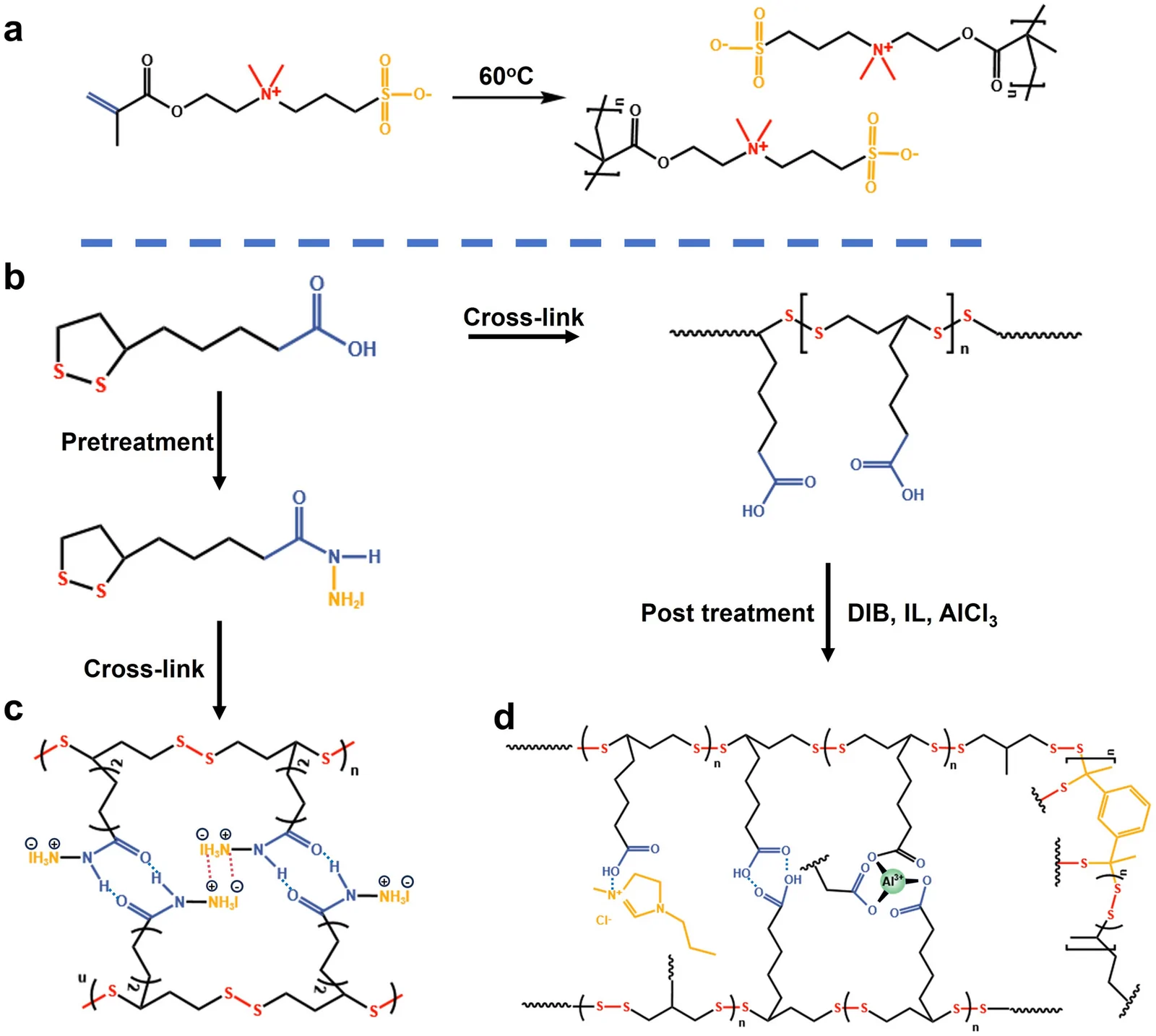
Chemical structure of representative SHPs based on thioctic acid or double bond **a** SBMA; **b** ɑ-Lipoic acid ring-opening polymerization [78]; **c** TA-NI [77]; and **d** ICE [84]

Monomers in this category typically feature electron-rich groups and dynamic bonds. For instance, Wang et al. incorporated the zwitterionic monomer 3-[[2-(methacryloyloxy)ethyl]dimethylammonio]propane-1-sulfonate (SBMA) and the initiator ammonium persulfate (APS) into perovskite precursor solutions (Fig. 13a) [92]. During perovskite annealing, SBMA monomers cross-linked, forming dynamic networks at grain boundaries. This conferred room-temperature self-healing capabilities to flexible PSCs (F-PSCs). Structurally, the positively charged ammonium group and the negatively charged sulfonate terminal group establish dynamic electrostatic interactions, facilitating ambient self-healing. Additionally, coordination between C=O/S=O groups and Pb^2+^ passivates defects.

In contrast to free radical polymerization, LA-based polymerization proceeds via dynamic disulfide bond exchange, involving three stages: First, under thermal, photolytic, or alkaline conditions, the cyclic disulfide bond (S–S) in LA cleaves, generating thiol groups (-SH) [121]. Then, thiol groups reversibly exchange with disulfide bonds, forming new S–S linkages while releasing thiol intermediates. Finally, the reaction achieves equilibrium, integrating disulfide bonds into the polymer backbone to enhance self-healing. The carboxyl groups in LA further contribute to reactivity (Fig. 13b). Additional functional groups can be incorporated, typically through either pre-cross-linking or post-cross-linking modification approaches. The pre-cross-linking method involves the direct reaction with carboxylic acids in LA to introduce functional groups prior to ring-opening polymerization. For instance, Li et al. synthesized a (5-(1,2-dithiolan-3-yl) pentanehydrazide hydroiodide (TA-NI) monomer from LA, which undergoes cross-linking at 80 °C (Fig. 13c) [77]. When incorporated into perovskite films, the iodinated quaternary ammonium salt coordinates with PbI_2_ to passivate defects while simultaneously imparting self-healing capabilities.

The post-cross-linking approach refers to the introduction of new functional groups after ring-opening polymerization. A representative example is the work by Song et al., who developed an ionic conductive elastomer (ICE) through post-polymerization modification of poly(LA), as shown in Fig. 13d [84]. This approach successfully integrated the high conductivity of ionic liquids with dynamic disulfide bonds, hydrogen bonds, and metal coordination bonds into the perovskite structure. The modified material effectively regulates crystallization processes, passivates grain boundary defects, and reduces potential differences between perovskite grains and boundaries. These modifications facilitate charge carrier extraction and transport at grain boundaries while enabling room-temperature repair of boundary cracks.

#### Synthesis Strategies of SHPs Based on Copolymers

**Fig. 14:**
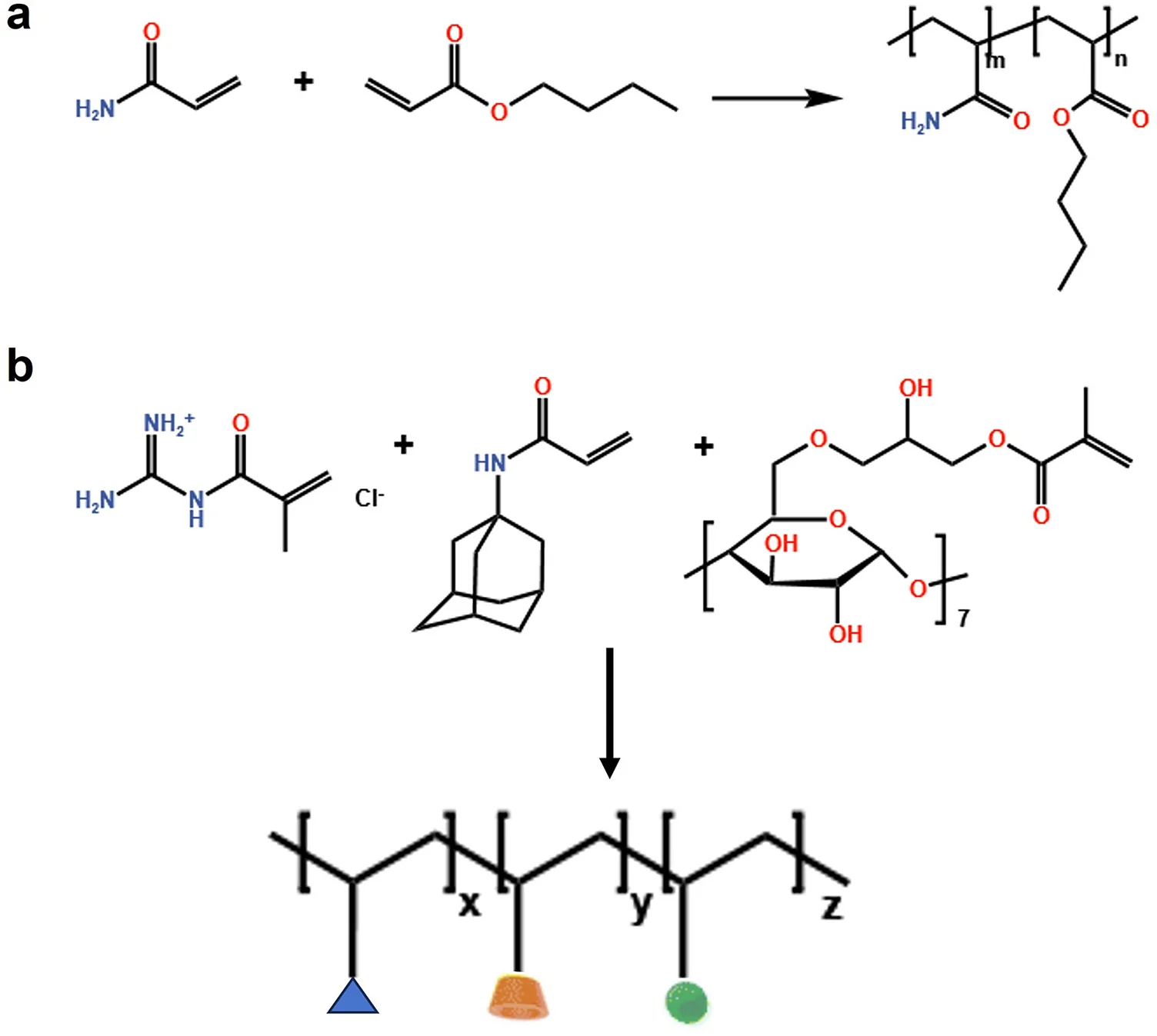
Chemical structure of representative SHPs based on copolymer **a** AD-23; **b** TPP

Copolymers, formed through copolymerization of two or more distinct monomers, exhibit multifunctional characteristics arising from the sequential arrangement of different monomeric units within their molecular chains [124]. The fundamental advantage of copolymers lies in their structural tunability, enabling precise regulation of material properties including hydrophobicity, electrical conductivity, and mechanical strength through judicious selection of monomer types and ratios. This approach facilitates synergistic integration of advantageous properties from distinct monomers (e.g., combining polyurethane’s dynamic reparability with PDMS’ hydrophobicity). Table 3 demonstrates self-healing performance and photovoltaic parameters of representative SHPs based on copolymer. Figure 14 demonstrates two representative copolymer systems. In Fig. 14a, Dong et al. synthesized copolymer AD-23 through free radical random copolymerization of acrylamide (AM) and n-butyl acrylate (BA) [125]. The copolymerization enabled precise regulation of glass transition temperature (*T*_g_) while introducing ester groups as hydrogen-bond acceptors along the polymer backbone. The resultant hydrogen-bonding interactions endowed F-PSCs with self-healing functionality under 70 °C thermal treatment.

**Table 3:** Self-healing performance and photovoltaic parameters of representative SHPs based on copolymer

Self-healing polymer	introducing strategy	SHPs action site	Dynamic bonds	Functional group	Self-healing conditions	Self-healing efficiency (%)	Device structure	PCE (%)	Stability	Refs.
AD-23	In	Additive engineering	Hydrogen bond	Amino and carbonyl group	70 °C, 5 min	Crack repair	PEN/ITO/PTAA/Perovskite(AD-23)/C_60_/BCP/Cu	21.99	85 °C 85%RH 260 h 80% PCE_initial_	[125]
TPP	In	Additive engineering	host–guest interaction, Hydrogen bond	Methacrylguanidine	60–70% RH	90	FTO/SnO_2_/Perovskite(TPP)/Spiro-OMeTAD/Ag	23.06	60–70%RH 1000 h 87% PCE_initial_	[126]
PU-PDMS-IU	Ex	Additive engineering	Hydrogen bond	Carbonyl group	100 °C, 10 min	93	PEN/PEDOT:PSS/PTAA/Perovskite(PU-PDMS-IU)/PC_61_BM/BCP/Au	23.25	85 °C N_2_ 1000 h 88% PCE_initial_	[85]
SMPU	Ex	Additive engineering	Hydrogen bond	Carbonyl group	37 °C, 30 min	80	PEN/ITO/SnO_2_/Perovskite(SMPU)/Spiro-OMeTAD/Au	21.33	25 °C N_2_ 3000 h 90% PCE_initial_	[127]
CT-TiO_2_	Ex	Interface Engineering	Hydrogen bond	Hydroxyl group	Water	100	FTO/c-TiO_2_/CT-TiO_2_/Perovskite/CIS/Au	10.41	25–30 °C 30–45%RH 2000 h 98% PCE_initial_	[95]
PU	Ex	Encapsulation	Hydrogen bond	Carbonyl group	RT, 24 h	99	PU5/ITO/NiO_x_/Perovskite/PC_61_BM/Ag	24.95	Unmentioned	[104]

Gao et al. further advanced this concept by incorporating a ternary prepolymer (TPP) system comprising methacrylguanidine hydrochloride (MAGH), glycidyl methacrylate-bonded *β*-cyclodextrin (GMA-CD), and N-adamantylacrylamide (N-AA) into perovskite precursor solutions (Fig. 14b) [126]. During annealing, ternary random copolymerization occurred, where N-AA and GMA-CD introduced host–guest interaction-based self-healing capability into perovskite films. This innovation conferred humidity-responsive self-repairing properties to 2D/3D PSCs. Concurrently, MAGH enhanced photovoltaic performance through chemical interactions with PbI_2_.

### Application-Oriented Design Strategies for SHPs

Building on the systematic review of the chemical foundations and integration methods of various SHPs in preceding sections, this section proposes a rational design strategy framework that is driven by application scenarios and structured around healing stimuli. Central to this framework is a shift from single-parameter optimization toward a synergistic design balancing target functionality, material properties, and process compatibility, thereby addressing the complex, multimodal failure modes encountered by PSCs in practical applications.

#### Healing Stimulus-Based Design Strategies

Given the complex operating environment of perovskite photovoltaic devices involving light, bias, and temperature variations, the activation of self-healing must balance repair efficiency with operational continuity. Precise selection of dynamic bonds is essential, leading to two principal design strategies—autonomous and stimulus-responsive—based on the severity, location, and nature of the damage.

Autonomous healing designs enable spontaneous repair under damage occurrence or normal operating conditions. This approach suits gradual, microscale damage such as ion migration, grain boundary microcracks, and interfacial delamination. The design relies on utilizing endogenous stimuli generated during device operation or engineering chemical systems with intrinsically high dynamics at room temperature. Specifically, the heat generated during device operation under illumination (typically 50–85 °C) can be harnessed to activate efficient bond exchange or recombination by precisely tuning the activation energy of dynamic covalent bonds or supramolecular interactions within this temperature window. For instance, dynamic covalent bonds with activation energies below 60–80 kJ mol^−1^ can be effectively activated under such conditions. Alternatively, in reversible systems based on boronic esters or hydrazone bonds, trace environmental moisture can serve as a healing trigger. The key lies in balancing the material’s hygroscopicity and water resistance to enable moisture-driven repair without degrading the perovskite layer. Another strategy involves incorporating photothermal moieties into the polymer network to convert visible light directly into localized heat during operation, thereby triggering thermally responsive dynamic bonds. A more direct method employs supramolecular interactions such as ionic bonds, multiple hydrogen bonds, or coordination bonds, whose inherent high chain mobility enables immediate autonomous healing at room temperature.

Stimulus-responsive healing is suited to sudden, macroscopic damage such as encapsulant cracking, electrode detachment, or large-area interfacial delamination. In this strategy, healing occurs only upon application of a specific external stimulus, offering controllability and on-demand functionality while avoiding unnecessary energy dissipation or side effects from persistent material dynamics. The design focuses on dynamic chemical systems sensitive to mild, targeted external stimuli to ensure efficient repair with minimal impact on other device components. Thermal triggering is a versatile approach, employing dynamic networks with higher activation energies to maintain stability at room temperature while enabling rapid repair under mild external heating. Light-triggered strategies offer exceptional spatiotemporal control and can be divided into near-infrared photothermal triggering and ultraviolet/visible photochemical triggering. The former introduces photothermal agents into the polymer to selectively convert near-infrared light into localized heat, enabling precise, localized repair with minimal thermal impact on the device bulk. The latter utilizes specific wavelengths to directly cleave or reform photoreversible bonds, permitting healing without thermal history at ambient temperature. Additional approaches include pressure-triggered designs based on mechanochemically responsive bonds, which convert mechanical force directly into a chemical driving force for repair, as well as pH- or chemical environment-triggered systems responsive to perovskite degradation by-products, thereby imparting environmental sensing and intelligent response capabilities.

A summary of key dynamic bonds and their characteristics pertinent to PSCs application is provided in Table 4.

**Table 4:** The key dynamic bonds and their characteristics pertinent to PSC application

Dynamic bond/interaction type	Representative examples	Primary healing trigger	Typical activation energy (*E*_*a*_) Condition (kJ mol^−1^)	Key features and application considerations in PSCs	Refs.
Diels–Alder reaction	Furan/maleimide adduct	Thermal (Retro: 100–150 °C; Reformation: 60–90 °C)	80–120	Two-temperature control. Suitable for off-line healing of encapsulation layers due to relatively harsh conditions	[128, 129]
Disulfide bonds	-S–S- exchange	Thermal/UV Light/Base Catalysis	30–60	Multi-responsive. Requires careful selection of photosensitizers for light triggering. Potential redox activity must be considered for interfacial layers	[130, 131]
Boronic ester bonds	Phenylboronic acid and diol	Moisture/Thermal/pH	40–70	Autonomous healing leveraging ambient humidity. Hydrophobicity must be tuned to prevent water ingress	[132, 133]
Transesterification	-COO- exchange with -OH/-NH-	Thermal (often with catalyst)	60–100	Effective for robust encapsulation. Catalysts are often required to lower operational temperature	[134, 135]
Imine bonds	-C = N-	Thermal/Acid/Humidity	50–80	Strong dynamics and pH responsiveness. Can be designed to respond to amine by-products from perovskite decomposition	[136]
Hydrogen bond	UPy, quadruple H-bonding	Thermal/Solvent Vapor	5–60	High dynamics enabling room-temperature autonomy. Ideal for additives and interface layers for real-time healing. May weaken in polar environments	[137, 138]
Ionomeric interaction	Poly(ionic liquid), ion pairs	Thermal/Solvent	50–300	Combines healing with potential ion conduction. Ion concentration must be controlled to avoid charge screening	[139]
Host–guest interaction	Crown ether/ammonium, cyclodextrin/adamantane	Thermal/Competitive Guest	10–100	Highly reversible and biocompatible. Requires precise molecular design for integration	[140, 141]
π-π stacking interaction	Molecules with Large Plane π Conjugate System	Thermal/Solvent	5–50	Highly reversible. Requires precise molecular design for integration	[142]
Metal coordination interaction	Zn^2+^-imidazole, Fe^3+^-carboxylate	Thermal/Light/Chemical	50–300	Strength and dynamics are tunable. Can anchor migrating Pb^2+^ ions, offering combined healing and passivation	[143]

#### Design Strategies for Specific Integration Scenarios

The design of SHPs must be deeply coupled with the physicochemical environment and dominant failure mechanisms inherent to their intended integration sites within the device. This section systematically analyzes the core damage modes at three critical locations: the bulk/grain boundaries, interfaces, and encapsulation layer, and derives targeted molecular design strategies.

The first scenario involves incorporating SHPs as additives within the perovskite bulk/grain boundaries. The primary damage modes here are mechanical (microcracks from bending, stretching, or impact in flexible devices) and chemical (ionic defects such as uncoordinated Pb^2+^ and I⁻ vacancies, as well as localized chemical degradation induced by moisture, oxygen, and light). Accordingly, effective SHP design must achieve three objectives: enhancing fracture toughness to suppress crack propagation; enabling dynamic and sustainable passivation of defect sites; and ensuring minimal disruption to the formation of high-quality perovskite crystals or to charge transport. Strategies to improve toughness rely on high polymer chain diffusivity and rapid reversibility of dynamic bonds. This involves selecting flexible chain segments with a low glass transition temperature (e.g., polyethers, polysiloxanes) as the matrix, incorporating high-density, low-activation-energy dynamic non-covalent bonds or room-temperature-responsive dynamic covalent bonds, and integrating a high density of Lewis base groups (e.g., carbonyl C=O, amino –NH_2_, phosphonic acid –PO_3_H_2_) for defect anchoring. To ensure good solubility and dispersion within the perovskite precursor solution and avoid phase separation, polymer molecular weight must be carefully controlled. An in situ polymerization strategy is preferred to guarantee uniform distribution along grain boundaries.

The second scenario focuses on designing SHPs as interfacial layers, aiming to construct a dynamically stable and efficient charge transport bridge. The interface between the perovskite and charge transport layers is a critical region for performance degradation, suffering from recombination losses due to interfacial defects, delamination risks from mismatched coefficients of thermal expansion, and impeded charge extraction due to energy-level offsets. To address these issues, interfacial SHPs must form strong yet reversible bonds with both adjacent materials, effectively passivate interfacial defects, and establish or maintain efficient charge transport pathways. The corresponding molecular design strategy emphasizes the introduction of a high density of strong anchoring groups (e.g., carboxyl, phosphonic acid) coupled with suitable dynamic bonds to enable structural reconfiguration. Furthermore, the polymer thickness must be precisely controlled, or the material itself should incorporate conjugated units or doped conductive nanomaterials to minimize series resistance.

The final scenario entails the use of SHPs as encapsulation layers. Outdoor exposure to rain, snow, hail, and sand can cause physical scratches or impact cracks, while temperature cycling induces stress leading to delamination between the encapsulant and the device. Such damage compromises barrier integrity, opening pathways for moisture/oxygen ingress and lead ion leaching. Consequently, SHPs for encapsulation must possess excellent and durable intrinsic barrier properties, along with the ability to rapidly and actively seal cracks upon damage. Additional requirements include strong adhesion to device surfaces, high optical transparency, and the capability to immobilize any potentially leached lead ions. The key functional design elements are: 1) Ultra-high barrier properties: The polymer backbone should incorporate hydrophobic segments (e.g., PDMS, fluorocarbon chains) to provide intrinsically low water vapor and oxygen transmission rates. 2) Strong adhesion and cohesion: Functional groups such as epoxies or silane coupling agents ensure robust bonding to glass/backsheets, while the network itself must exhibit high strength. 3) Lead immobilization capacity: Integration of lead-capturing groups (e.g., carboxylic acid, sulfonic acid, crown ethers) enables a “leak-and-capture” safety mechanism. 4) Controllable triggered healing: Employing robust, stable dynamic covalent networks (e.g., based on Diels–Alder bonds) ensures reliable repair.

A summary of functional group optimization and their characteristics pertinent to PSCs application is provided in Table 5.

**Table 5:** The functional group optimization and their characteristics pertinent to PSCs application

Functional group/unit	Primary mechanism	Optimization effects	Refs.
Carboxyl group (–COOH)	Forms strong coordination bonds with uncoordinated Pb^2+^; modulates crystallization kinetics	Passivates deep-level traps, improves film uniformity, enhances V_OC_ and FF	[144]
Amino group (–NH_2_, –NHR)	Coordinates with Pb^2+^ or forms H-bonds with I⁻; passivates defects and controls crystal growth	Suppresses non-radiative recombination, inhibits ion migration, improves stability	[145]
Sulfonic acid group (–SO_3_H)	Strong coordination with Pb^2+^ via acidic protons; improves interfacial wetting	Effective defect passivation, enhanced PCE, improved moisture tolerance	[146]
Carbonyl group (–C=O)	Moderate coordination with Pb^2+^; acts as a hydrogen-bond acceptor	Increases charge carrier lifetime, improves device reproducibility	[147]
Fluorine/Fluorinated groups (–F, –CF_3_)	Provides hydrophobicity; tunes energy levels via high electronegativity	Significantly boosts environmental stability, reduces energy loss at interfaces	[148]
Long Alkyl/Aromatic Chains	Forms hydrophobic barriers; modulates polymer aggregation and interfacial energy	Enhances moisture resistance, improves film morphology and charge extraction	[149]
Quaternary Ammonium/Ionic groups	Suppresses ion migration via electrostatic shielding; forms interfacial dipoles	Reduces hysteresis, improves operational stability, modifies energy alignment	[150]
Epoxy/Cross-linking groups	Forms cross-linked networks upon light/heat exposure, encapsulating the perovskite	Enhances mechanical robustness and moisture resistance; inhibits delamination	[151]
Phosphonic acid group (–PO_3_H_2_)	Strong bilateral anchoring to both Pb^2+^ and metal oxide substrates (e.g., TiO_2_)	Reduces interfacial recombination, improves electron extraction and adhesion	[152]
Selenophene/Tellurophene units	Stronger electron-donating ability and better orbital overlap than thiophene	Enhances hole mobility in HTLs, reduces voltage loss via better energy matching	[153]
UV-Absorbing/Converting groups	Absorbs harmful UV photons and converts them to heat or visible light	Protects perovskite from UV-induced degradation	[154]
Ionic liquid polymer units	Combines ionic conductivity (from side chains) with polymeric stability	Effectively suppresses ion migration, eliminates hysteresis, aids charge extraction	[155]
Porous framework structure	Provides nano-confinement with well-defined pore size and chemical environment	Directs oriented perovskite crystallization; physically isolates grains to suppress ion migration	[156]
Host–guest inclusion units	Encapsulates perovskite precursors or organic cations via hydrophobic cavities	Modulates crystallization kinetics; stabilizes organic components against volatilization	[157]

## Conclusion and Outlook

The integration of SHPs with PSCs has opened new avenues to address the instability of perovskite materials and the environmental vulnerability of devices. In this review, we systematically elucidate the self-healing mechanisms of self-healing elastomers. Furthermore, we emphasize the synergistic effects of multiple dynamic bonds, demonstrating their capability to enable autonomous repair of PSCs under mild conditions. The underlying principles of self-healing in PSCs under both chemical and mechanical damage modes are comprehensively discussed. A multi-scale evaluation framework for self-healing performance is proposed, encompassing macroscopic device performance testing, microscopic structural and morphological characterization, as well as chemical composition and structural analysis. Besides, the multifunctional roles of SHPs—as additives, interfacial modifiers, and encapsulants in PSCs—are systematically evaluated, with their performance contrasted against conventional polymeric systems. Beyond defect passivation, crystallization regulation, and mechanical flexibility enhancement, SHPs uniquely empower PSCs with self-healing capabilities—autonomously sealing microcracks, restoring operational stability, and mitigating lead leakage to minimize environmental risks. Notably, two primary strategies for incorporating SHPs into PSCs are discussed: ex situ polymerization (pre-synthesized polymers blended into precursors) and in situ polymerization (monomers polymerized during perovskite annealing). Application-oriented design principles for SHPs are systematically examined through representative case studies, with particular emphasis placed on structure–property relationships optimized for photovoltaic durability. Although self-healing polymers offer a highly promising solution for achieving long-term stability in PSCs, they face the following core challenges compared to classical one-time passivation strategies:

1. Inadequate Understanding of Fundamental Mechanisms and Long-Term Reliability

The application of self-healing polymers in perovskite photovoltaics is relatively recent, and a comprehensive theoretical framework for their repair mechanisms under various stimuli such as light, heat, and electrical bias remains underdeveloped. Furthermore, most studies have only demonstrated single or a limited number of repair cycles, with a notable lack of reported research on the sustained healing cyclability of these materials over a device’s full operational lifetime. This deficiency in theoretical understanding and the uncertainty surrounding long-term cyclic stability are key factors limiting their industrial adoption. Therefore, future research must focus on an in-depth investigation of in situ repair mechanisms by utilizing advanced in situ characterization techniques to observe the self-healing process in real-time under actual working conditions (e.g., illumination, heat, bias). This will help clarify the chemical and structural evolution at the polymer–perovskite interface before and after repair, providing a theoretical foundation for material design. Additionally, it is essential to establish standardized protocols for long-term reliability assessment. Moving beyond short-term performance recovery demonstrations, accelerated aging test protocols for healing cycle lifetime must be developed to quantitatively evaluate the endurance limits of self-healing functionality under simulated real-world operating conditions.

(2) Complexity and Functional Trade-offs in Molecular Design

Endowing polymers with self-healing capabilities (e.g., by introducing dynamic covalent bonds or supramolecular interactions) is inherently a complex molecular engineering task. More critically, a delicate balance must be struck among the self-healing function, effective defect passivation capability, and suitable electrical conductivity. Currently, most self-healing polymers exhibit insulating properties to ensure effective repair and passivation, which may impede charge carrier transport and lead to losses in device fill factor and power conversion efficiency. Therefore, future research should focus on developing novel polymers or composite materials that integrate dynamic self-healability, effective defect-passivating sites, and excellent charge transport characteristics. An ideal self-healing polymer should enhance both the performance and flexibility of PSCs, while also enabling the devices to withstand dozens of healing cycles without significant degradation. For encapsulation materials, their water vapor transmission rate (WVTR) and oxygen transmission rate (OTR) must meet or exceed the stringent standards established for electronic device packaging.

(3) Scalable Fabrication and Process Compatibility

The foremost challenge in translating self-healing strategies from laboratory-scale demonstrations to industrial manufacturing lies in bridging the chasm between spin-coating-based processing and scalable, high-throughput fabrication techniques. Material-process compatibility presents a primary impediment. The rheological properties (e.g., viscosity, drying kinetics) of most SHPs, designed primarily for optimal healing, are often mismatched with the requirements of large-area, continuous deposition methods such as slot-die or blade coating. Developing coat-able ink formulations with broad processing windows, suitable rheology, and retained high performance is therefore a critical first step toward scalable manufacturing. In addition, cost-effective integration with continuous manufacturing processes, notably roll-to-roll (R2R) processing, constitutes the ultimate test for industrial adoption. This challenge extends beyond raw material economics to require that the entire system—including polymers, solvents, and process parameters—can adapt to high-speed, continuous, low-waste production rhythms. Addressing these hurdles demands a concerted effort integrating materials design, ink engineering, process development, and equipment design—a multidisciplinary integration essential for truly bridging laboratory innovation and industrial-scale fabrication.

(4) Data-Driven and Machine Learning-Accelerated Development

To navigate the complex multi-parameter design space, machine learning (ML) offers a transformative approach. The immediate task involves mining existing chemical (e.g., PolyInfo) and photovoltaic performance databases (e.g., Perovskite Database) to identify preliminary patterns. The future cornerstone is constructing specialized, high-quality databases linking polymer chemical descriptors, healing properties (efficiency, conditions), and device stability metrics. Such databases will empower ML models for high-throughput virtual screening of novel healable materials, predicting healing kinetics under various stresses, and optimizing device architectures. This data-driven paradigm will shift the field from empirical trials to intelligent, predictive design.
